# Supplementary figures and images for: The 3-phosphoinositide–dependent protein kinase 1 is an essential upstream activator of protein kinase A in malaria parasites
Source: PLoS Biol. 2021 Dec 8;19(12):e3001483. doi: 10.1371/journal.pbio.3001483 (PMC8687544; doi:10.1371/journal.pbio.3001483)

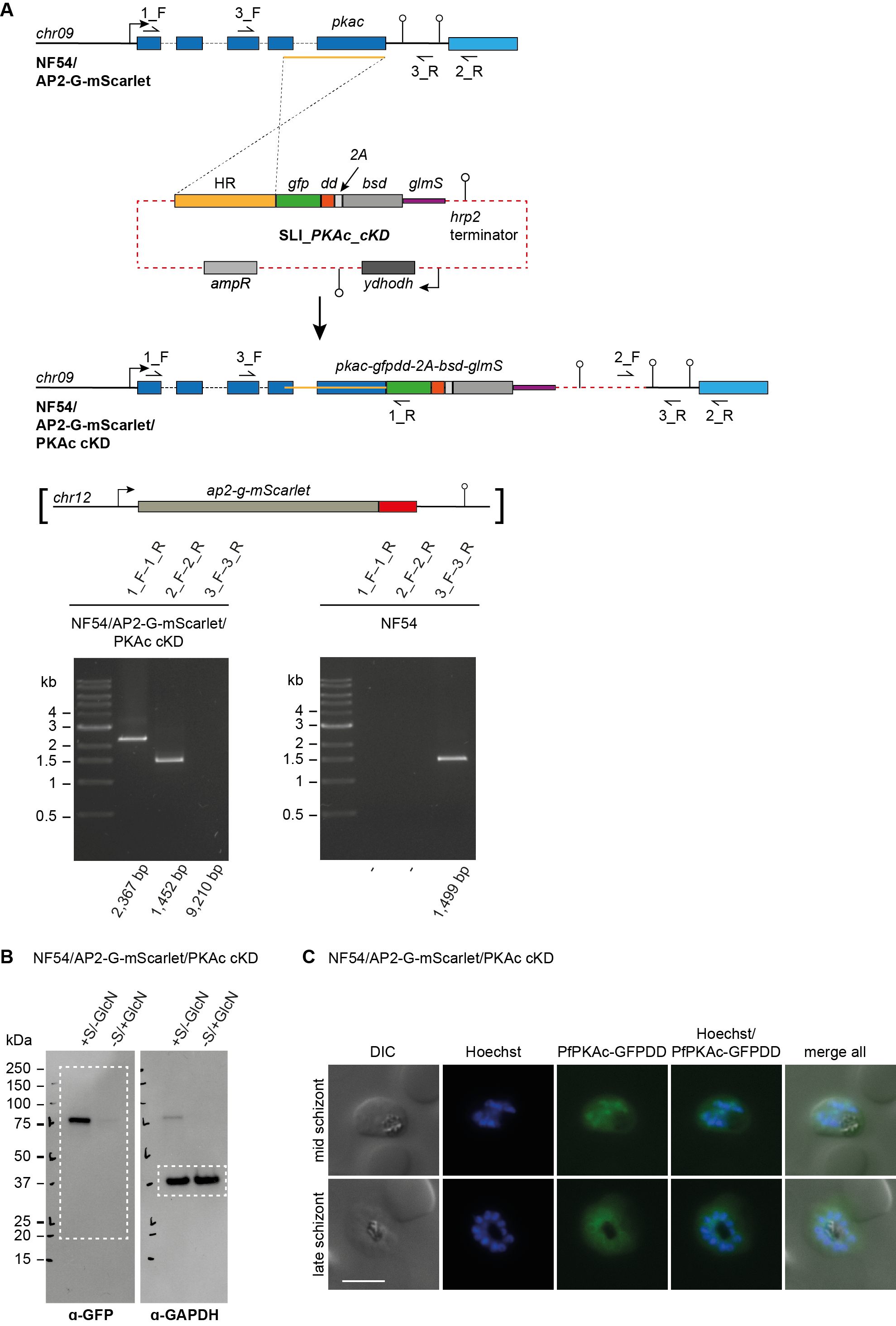

Supplement: S1 Fig — (A) Top: Scheme depicting the WT pfpkac locus, the SLI_PKAc_cKD construct transfected into NF54/AP2-G-mScarlet parasites and the edited pfpkac locus in NF54/AP2-G-mScarlet/PKAc cKD parasites. Primers used for diagnostic PCRs are indicated. Middle: The schematic map of the edited pfap2-g-mScarlet locus in the NF54/AP2-G-mScarlet parasite line [41] is shown in brackets. Bottom: Results of PCR reactions performed on gDNA of NF54/AP2-G-mScarlet/PKAc cKD and NF54 WT control parasites confirm correct gene editing. (B) Full size western blot showing expression of PfPKAc-GFPDD in late schizonts cultured under protein- and RNA-depleting (–Shield-1/+GlcN) and control conditions (+Shield-1/–GlcN). Lysates derived from an equal number of parasites were loaded per lane. The membrane was first probed with α-GFP followed by α-GAPDH control antibodies. MW PfPKAc-GFP = 67.3 kDa, MW PfGAPDH = 36.6 kDa. Dashed lines mark the blot sections shown in Fig 1A. (C) Expression of PfPKAc-GFPDD in mid and late schizonts under protein- and RNA-stabilising conditions (+Shield-1/–GlcN) as assessed by live cell fluorescence imaging. Parasites were previously synchronised to an 8-hour window and imaged at 32 to 40 hpi and 40 to 48 hpi. Representative fluorescence images are shown. Parasite DNA was stained with Hoechst. Scale bar = 5 μm. DIC, differential interference contrast; hpi, hours postinvasion; WT, wild-type. (TIF) [file pbio.3001483.s001.tif]

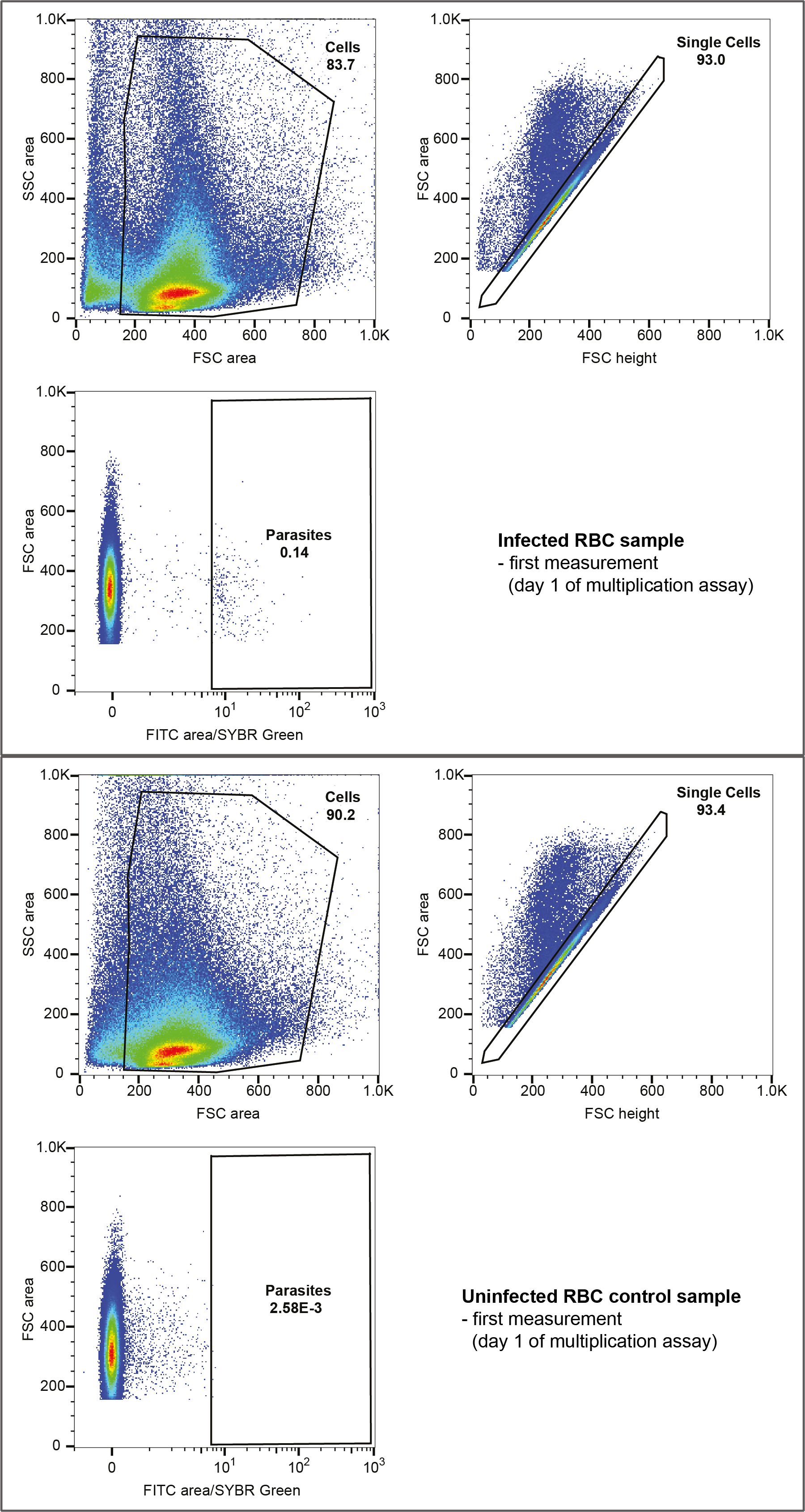

Supplement: S2 Fig — Representative flow cytometry plots of a parasite culture (top frame; NF54/AP2-G-mScarlet/PKAc cKD, +Shield-1/–GlcN) and an uninfected RBC control sample (bottom frame) on day 1 of the multiplication assay are shown. Events were consecutively gated for the expected cell size, singlets and infected (SYBR green positive) RBCs. The resulting parasite multiplication plots are shown in Figs 1, 2, and 4–6 and in S6, S9, and S11 Figs. RBC, red blood cell. (TIF) [file pbio.3001483.s002.tif]

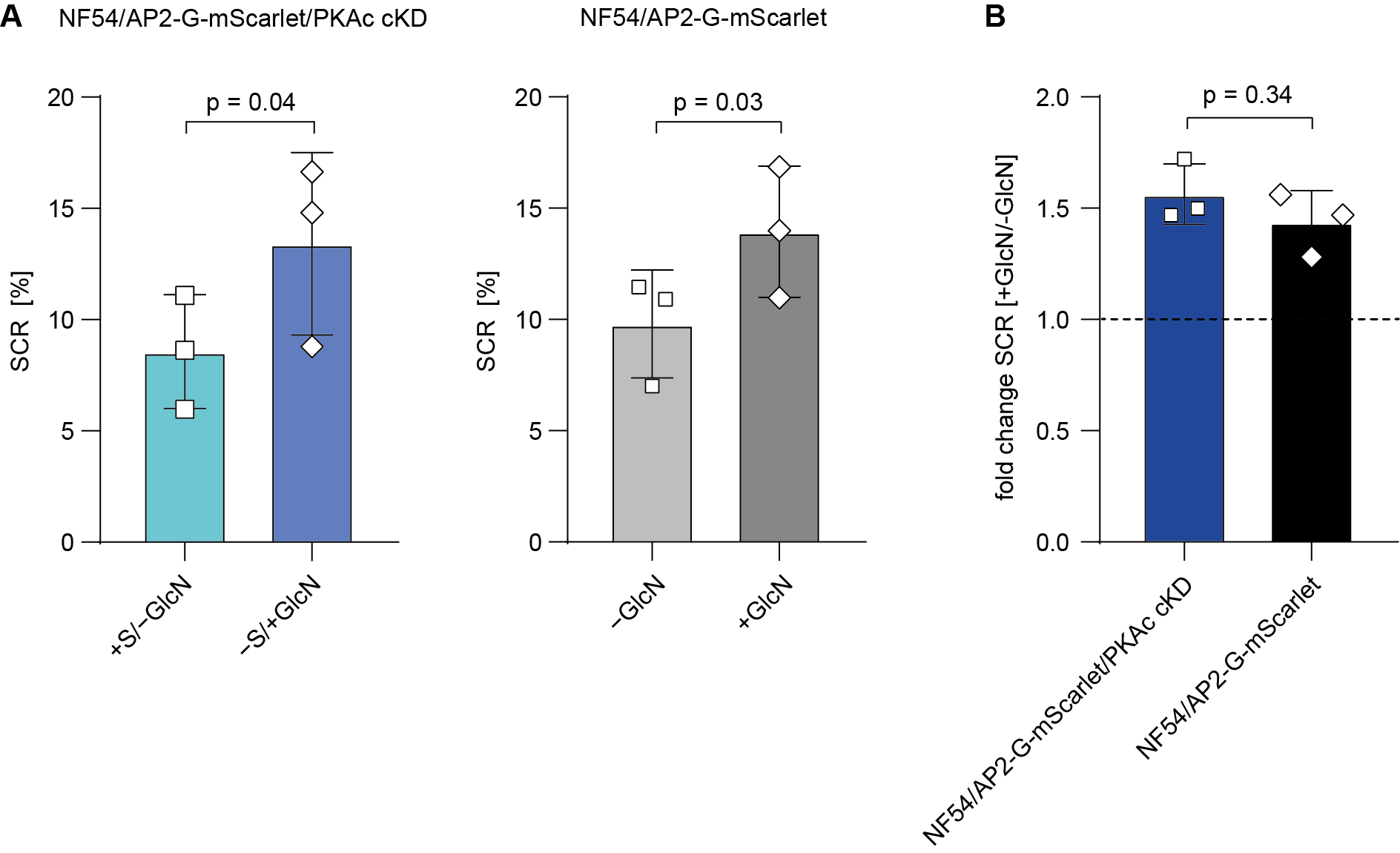

Supplement: S3 Fig — (A) Left panel: SCRs of NF54/AP2-G-mScarlet/PKAc cKD parasites cultured under protein- and RNA-depleting (–Shield-1/+GlcN) (turquoise) and control conditions (+Shield-1/–GlcN) (blue). Right panel: SCRs of NF54/AP2-G-mScarlet control parasites cultured in the presence (+GlcN) (light grey) or absence of GlcN (–GlcN) (dark grey). SCRs were determined by high content imaging and automated image analysis by assessing PfAP2-G-mScarlet positivity among the total number of Hoechst-stained iRBCs. For each experiment, at least 607 Hoechst-positive cells were assessed for PfAP2-G-mScarlet expression. Open squares represent data points for individual replicates and the means and SD (error bars) of 3 biological replicate experiments are shown. Differences in SCRs have been compared using a paired 2-tailed Student t test (statistical significance cutoff: p < 0.05). The raw data are available in the source data file (S2 Data). (B) Mean fold change in SCRs of NF54/AP2-G-mScarlet/PKAc cKD parasites cultured under–Shield-1/+GlcN compared to +Shield-1/–GlcN conditions (dark blue) and of NF54/AP2-G-mScarlet control parasites cultured under +GlcN compared to–GlcN conditions (black). Differences in the fold change in SCRs between NF54/AP2-G-mScarlet/PKAc cKD and NF54/AP2-G-mScarlet control parasites have been compared using an unpaired 2-tailed Student t test (statistical significance cutoff: p < 0.05). S, Shield-1; GlcN, glucosamine. The raw data are available in the source data file (S2 Data). iRBC, infected red blood cell; SCR, sexual commitment rate. (TIF) [file pbio.3001483.s003.tif]

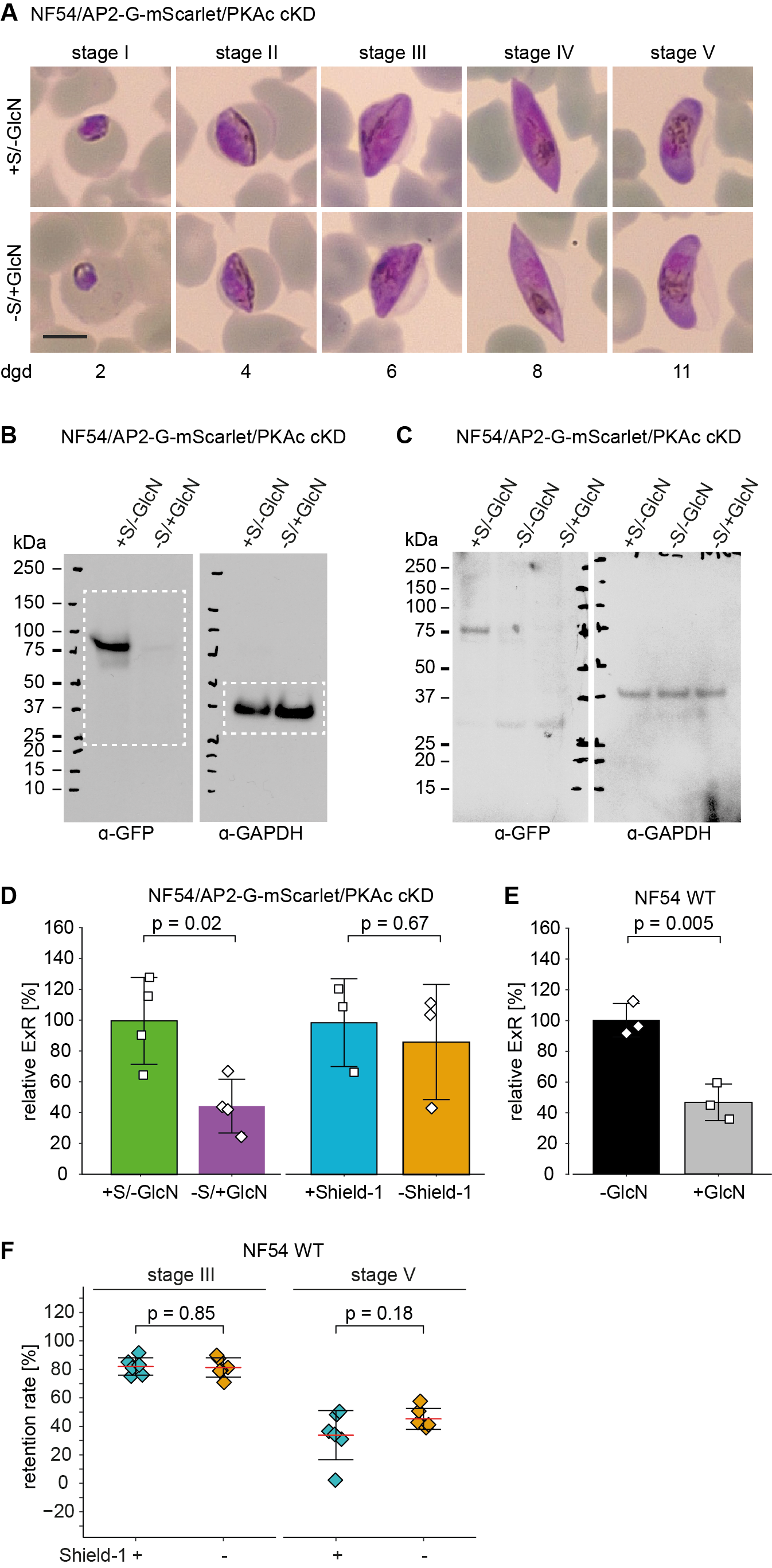

Supplement: S4 Fig — (A) Representative images captured from Giemsa-stained blood smears showing the distinct morphology of stage I to V gametocytes cultured under PfPKAc-GFPDD-depleting (–Shield-1/+GlcN) and control conditions (+Shield-1/–GlcN) over 11 days of maturation. Synchronous parasites were split (±Shield-1/±GlcN) as sexual/asexual ring stage parasites 24 hours after the induction of sexual commitment in the preceding IDC. To eliminate asexual parasites, gametocytes were cultured in +SerM supplemented with 50 mM GlcNAc from day 1 to 6 of gametocytogenesis. Scale bar = 5 μm. dgd, day of gametocyte development. (B) Full size western blot showing expression of PfPKAc-GFPDD in mature stage V gametocytes (day 11) under protein- and RNA-depleting (–Shield-1/+GlcN) and control conditions (+Shield-1/–GlcN). Lysates derived from an equal number of parasites were loaded per lane. The membrane was first probed with α-GFP followed by α-GAPDH control antibodies. MW PfPKAc-GFPDD = 79.8 kDa, MW PfGAPDH = 36.6 kDa. Dashed lines mark the blot sections shown in Fig 1D. (C) Full size western blot comparing expression of PfPKAc-GFPDD under protein- and RNA-depleting (–Shield-1/+GlcN), protein-depleting (–Shield-1/–GlcN) and control conditions (+Shield-1/–GlcN) in mature stage V gametocytes (day 11). Lysates derived from an equal number of parasites were loaded per lane. (D) Relative ExRs of NF54/AP2-G-mScarlet/PKAc cKD mature stage V gametocytes (day 14) cultured under protein- and RNA-depleting (–Shield-1/+GlcN) (purple) and control conditions (+Shield-1/–GlcN) (green) or under protein-depleting only (–Shield-1) (orange) and control conditions (+Shield-1) (blue). Open squares represent data points for individual replicates and the means and SD (error bars) of at least 3 biological replicate experiments are shown. Differences in ExRs have been compared using an unpaired 2-tailed Student t test (statistical significance cutoff: p < 0.05). The raw data are available in the source data file (S2 Data) [file pbio.3001483.s004.tif]

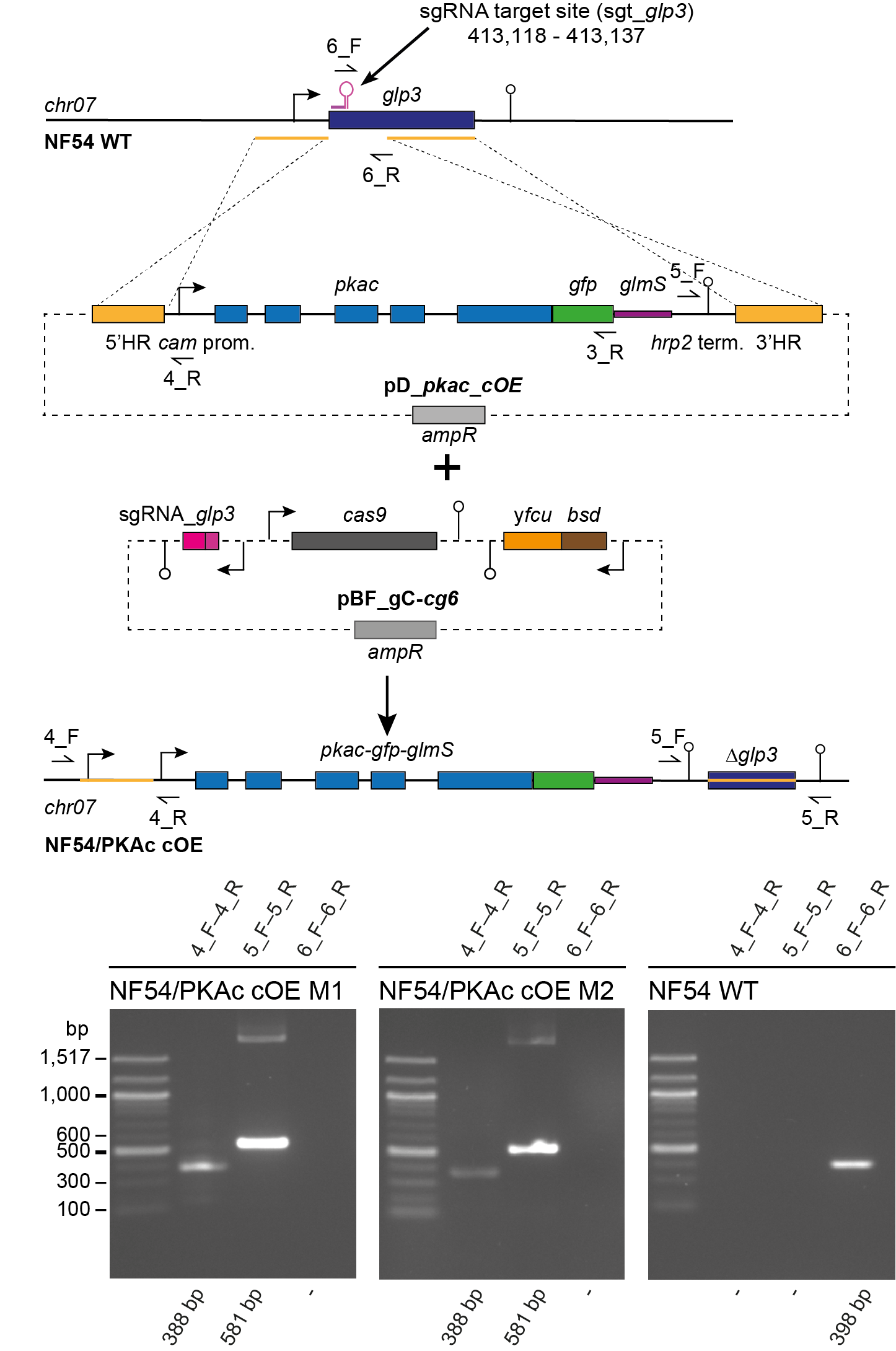

Supplement: S5 Fig — Top: Scheme depicting the WT glp3 target locus, the donor (pD_pkac_cOE) and pBF_gC-cg6 suicide constructs transfected into NF54 WT parasites to generate the NF54/PKAc cOE parasite line and the edited glp3 locus. Primers used for diagnostic PCRs are indicated. Bottom: Results of PCR reactions performed on gDNA of 2 clones (M1 and M2) of the NF54/PKAc cOE line and NF54 WT control parasites confirm successful insertion of the PfPKAc cOE cassettes into the glp3 locus. cOE, conditional overexpression; WT, wild-type. (TIF) [file pbio.3001483.s005.tif]

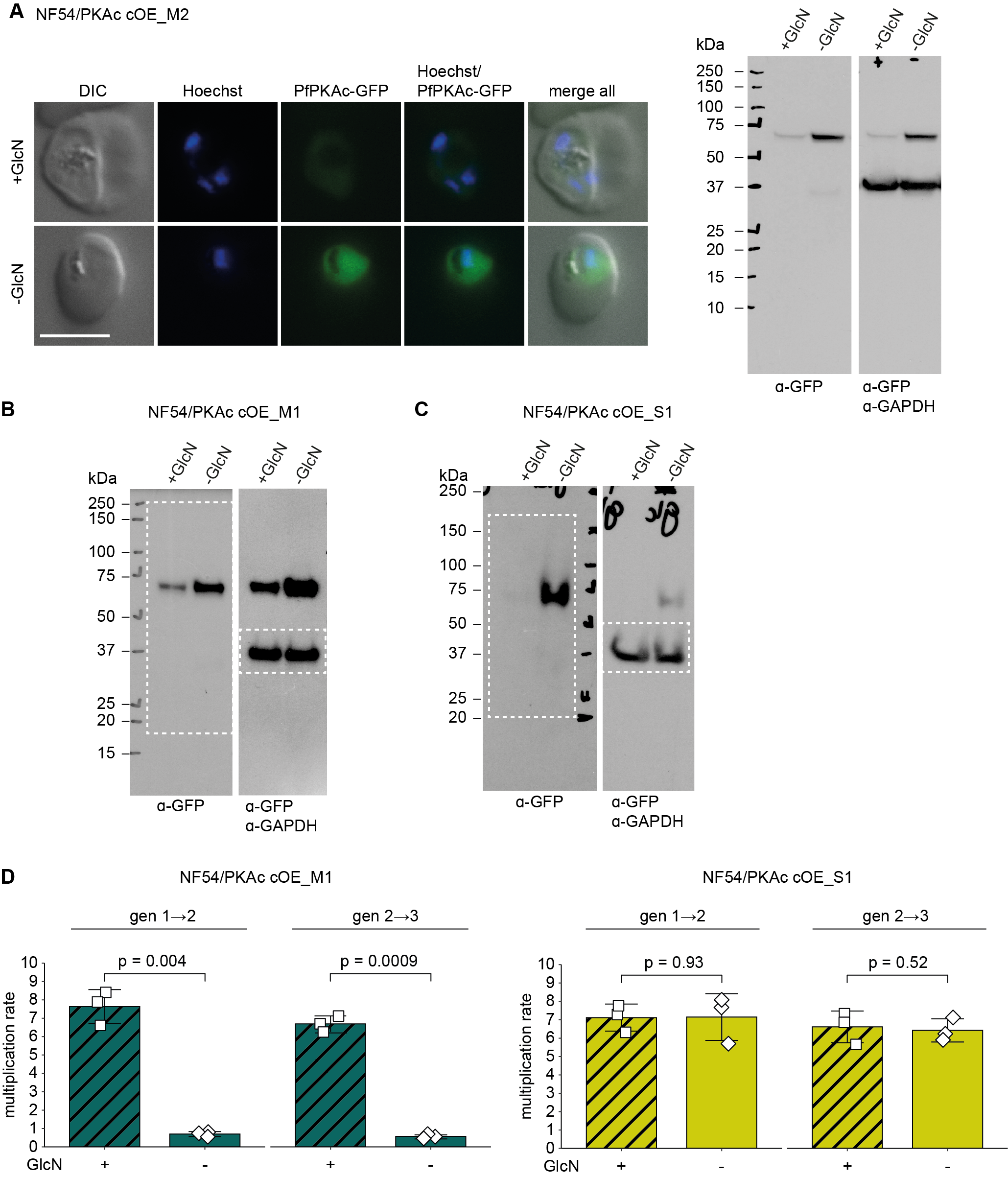

Supplement: S6 Fig — (A) Expression of PfPKAc-GFP in NF54/PKAc cOE M2 parasites under overexpression-inducing (–GlcN) and control conditions (+GlcN) as assessed by live cell fluorescence imaging and western blot analysis. Synchronous parasites (0 to 8 hpi) were split (±GlcN) 40 hours before sample collection. Representative fluorescent images are shown. Parasite DNA was stained with Hoechst. Scale bar = 5 μm. For western blot analysis, parasite lysates derived from equal numbers of parasites were loaded per lane. The membrane was first probed with α-GFP followed by α-GAPDH control antibodies. MW PfPKAc-GFP = 67.3 kDa, MW PfGAPDH = 36.6 kDa. The full size western blot is shown. (B) Full size western blot showing expression of PfPKAc-GFP in NF54/PKAc cOE M1 parasites under overexpression-inducing (–GlcN) and control conditions (+GlcN) conditions. Parasites were cultured and samples prepared as described in panel A. The membrane was first probed with α-GFP followed by α-GAPDH control antibodies. MW PfPKAc-GFPDD = 79.8 kDa, MW PfGAPDH = 36.6 kDa. Dashed lines mark the blot sections shown in Fig 2A. (C) Full size western blot shows expression of PfPKAc-GFP in NF54/PKAc cOE S1 parasites under overexpression-inducing (–GlcN) and control (+GlcN) conditions. Parasites were cultured and samples prepared as described in panel A. The membrane was first probed with α-GFP followed by α-GAPDH control antibodies. MW PfPKAc-GFPDD = 79.8 kDa, MW PfGAPDH = 36.6 kDa. Dashed lines mark the blot sections shown in Fig 2E. MW PfPKAc-GFP = 67.3 kDa, MW PfGAPDH = 36.6 kDa. (D) Parasite multiplication rates of NF54/PKAc cOE M1 (left) and S1 survivor parasites (right) under overexpression-inducing (–GlcN) and control conditions (+GlcN) over 2 generations. Open squares represent data points for individual replicates and the means and SD (error bars) of 3 biological replicates are shown. Differences in multiplication rates have been compared using a paired 2-tailed Student t test (statistical significance cutoff: p [file pbio.3001483.s006.tif]

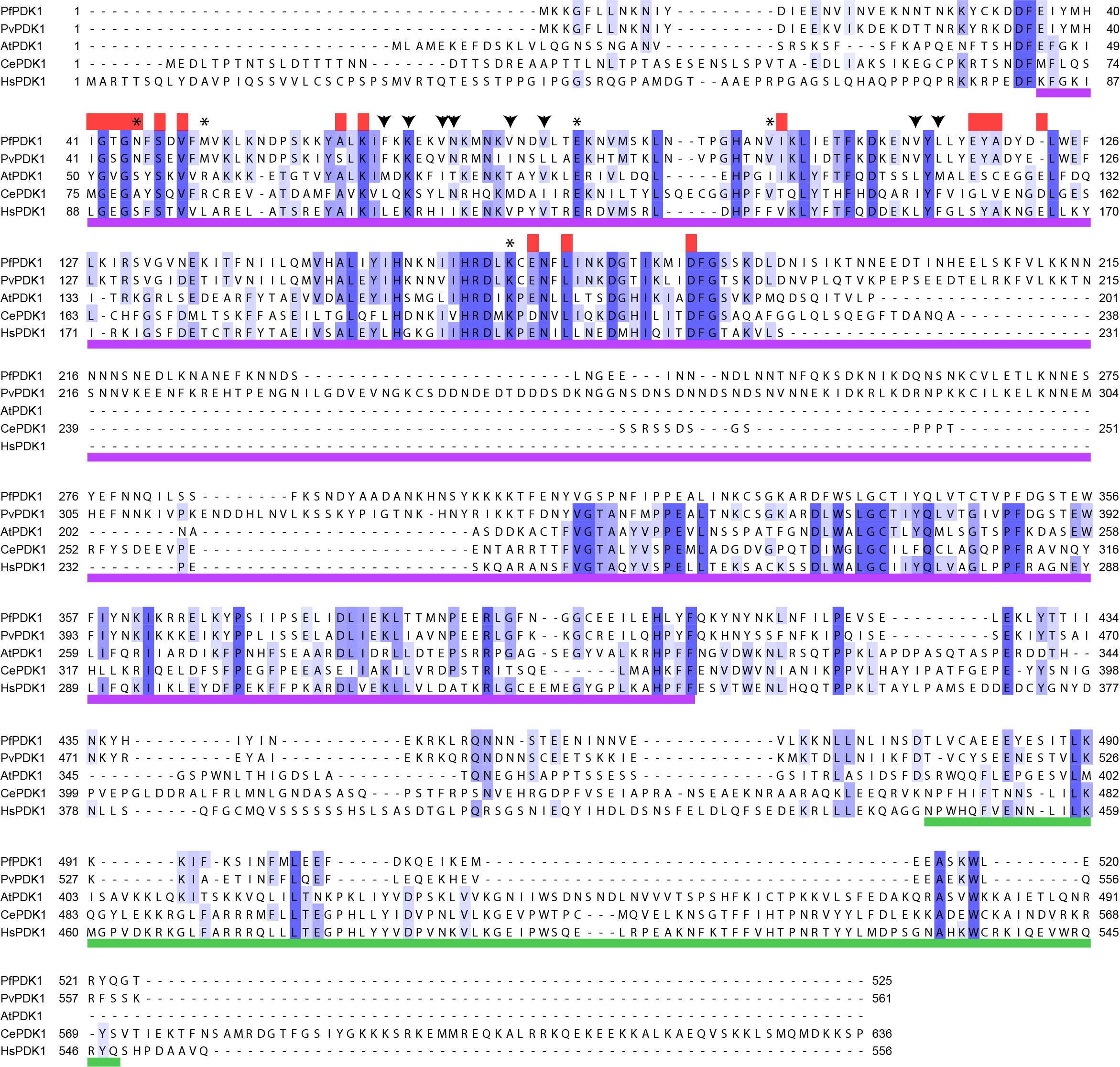

Supplement: S7 Fig — Clustal Omega [96] multiple sequence alignment of PfPDK1 (Pf3D7_1121900/UniProt ID Q8IIE7) and PvPDK1 (PVX_091715/UniProt ID A5K4N1) with well-characterised PDK1 homologues from Arabidopsis thaliana (UniProt ID Q9XF67), Caenorhabditis elegans (UniProt ID Q9Y1J3), and humans (UniProt ID O15530). Residues are highlighted in blue gradient depending on fractional conservation. The kinase catalytic domain spans residues 82 to 342 in human PDK1 [52] (purple section underneath the sequences), which also includes a carboxyl-terminal PH domain spanning residues 446–548 [51] (green section). Arrowheads denote residues forming the PIF-binding pocket in human PDK1 [22], asterisks denote residues mutated in PfPKAc OE survivors identified in this study and red bars denote residues that form part of the ATP-binding cleft. PH, pleckstrin homology. (TIF) [file pbio.3001483.s007.tif]

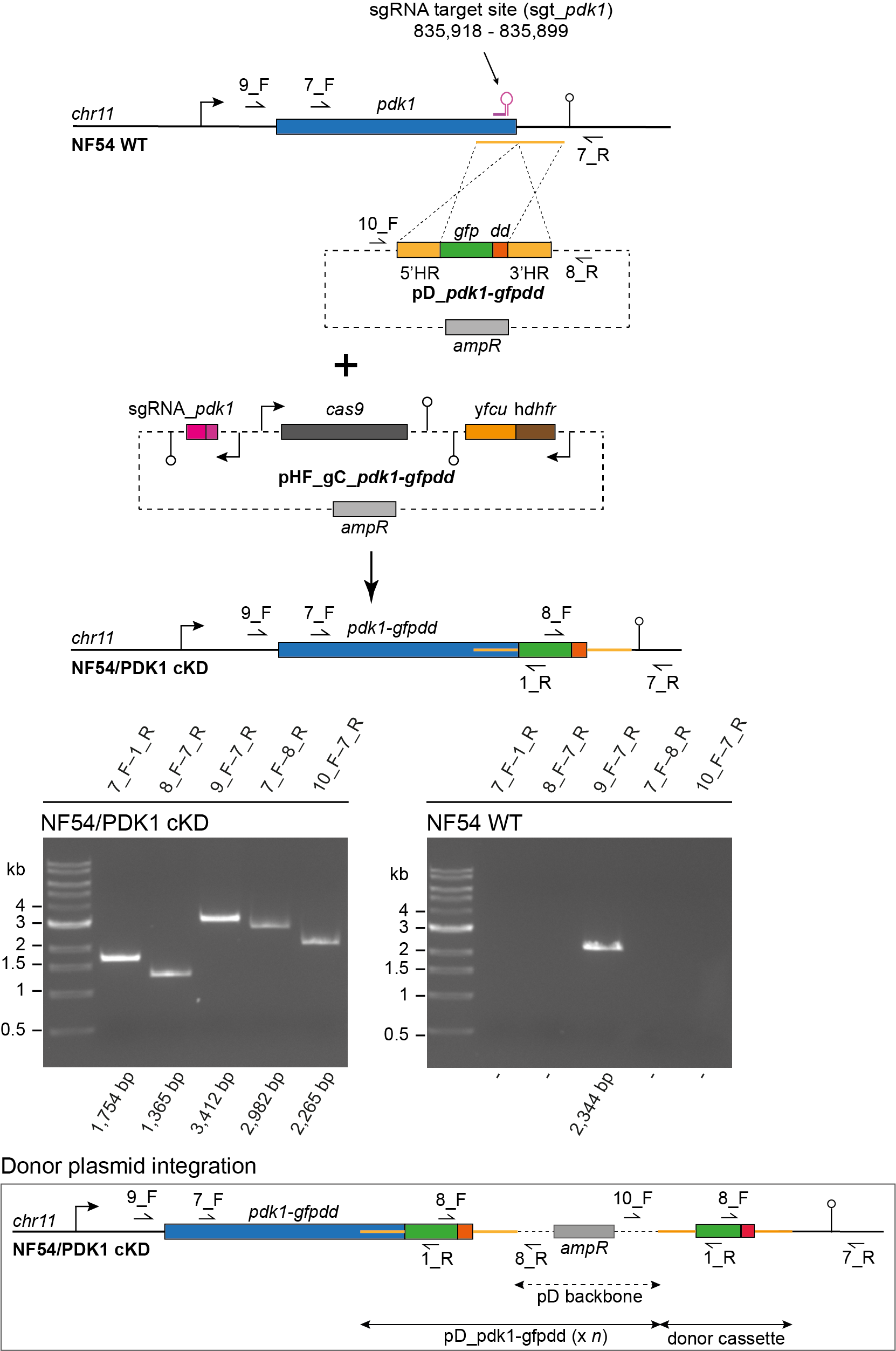

Supplement: S8 Fig — Top: Scheme depicting the WT pfpdk1 locus, the donor (pD_pdk1-gfpdd) and the suicide (pHF_gC_pdk1-gfpdd) constructs transfected into NF54 WT parasites to generate the NF54/PDK1 cKD parasite line and the edited pfpdk1 locus. Primers used for diagnostic PCRs are indicated. Middle: Results of PCR reactions performed on gDNA of NF54/PDK1 cKD and NF54 WT control parasites confirm correct editing of the pfpdk1 locus as well as plasmid concatemer integration. Bottom: Schematic map illustrating the integration of a donor plasmid concatemer based on double-crossover recombination of nonadjacent HRs on the concatemer. To simplify the schematic, the integration of a tandem assembly only is shown. HR, homology region; WT, wild-type. (TIF) [file pbio.3001483.s008.tif]

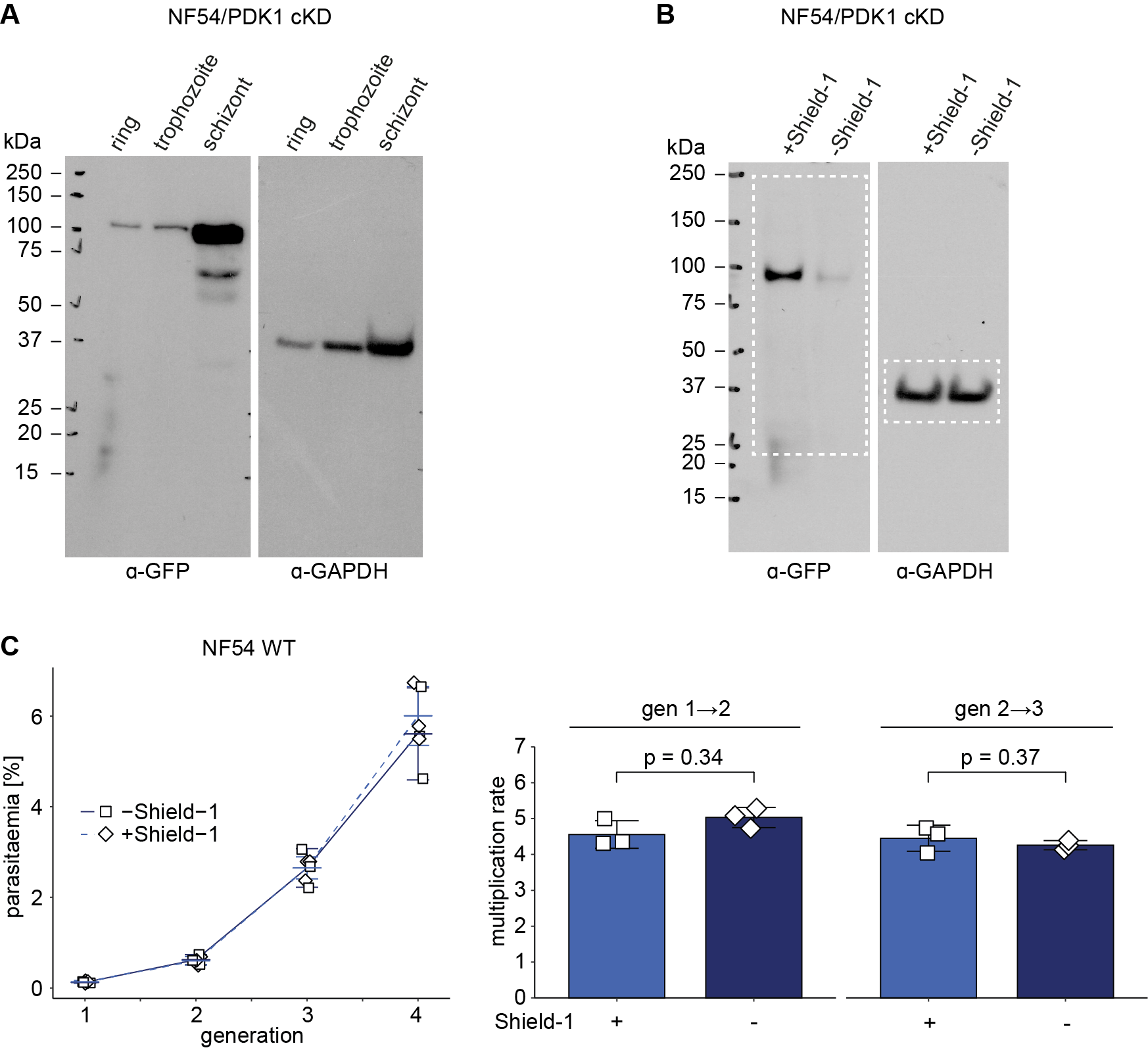

Supplement: S9 Fig — (A) Expression of PfPDK1-GFPDD in ring (18 to 24 hpi), trophozoite (24 to 30 hpi), and schizont (42 to 48 hpi) stages of NF54/AP2-G-mScarlet/PDK1 cKD parasites under protein-stabilising (+Shield-1) conditions as assessed by western blot analysis. Lysates derived from equal numbers of parasites were loaded per lane. The membrane was first probed with α-GFP followed by α-GAPDH control antibodies. MW PfPDK1-GFPDD = 101.1 kDa, MW PfGAPDH = 36.6 kDa. The full size western blot is shown. (B) Full size western blot shows expression of PfPDK1-GFPDD in NF54/AP2-G-mScarlet/PDK1 cKD parasites under protein-depleting (–Shield-1) and control (+Shield-1) conditions. Synchronous parasites (0 to 8 hpi) were split (±Shield-1) 40 hours before collection of the samples. Lysates derived from equal numbers of parasites were loaded per lane. The membrane was first probed with α-GFP followed by α-GAPDH control antibodies. MW PfPDK1-GFPDD = 101.1 kDa, MW PfGAPDH = 36.6 kDa. Dashed lines mark the blot sections shown in Fig 4B. (C) Increase in parasitaemia (left) and corresponding parasite multiplication rates (right) of NF54 WT parasites cultured in presence (+Shield-1) and absence of Shield-1 (–Shield-1). Synchronous parasites (0 to 6 hpi) were split (±Shield-1) 18 hours before the first measurement in generation 1. Open squares represent data points for individual replicates and the means and SD (error bars) of 3 biological replicates are shown. Differences in multiplication rates have been compared using a paired 2-tailed Student t test (statistical significance cutoff: p < 0.05). The raw data are available in the source data file (S2 Data). hpi, hours postinvasion; WT, wild-type. (TIF) [file pbio.3001483.s009.tif]

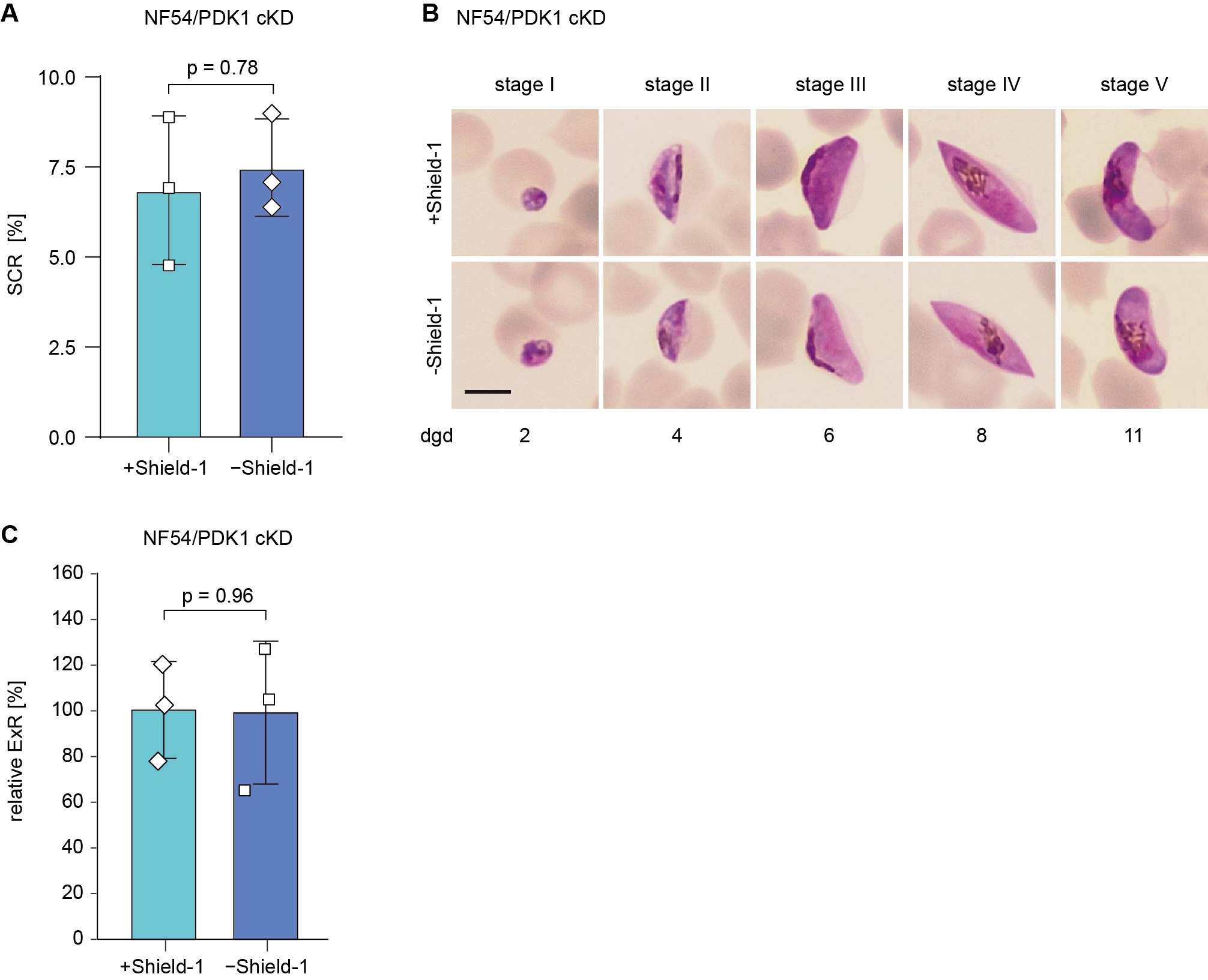

Supplement: S10 Fig — (A) SCRs of NF54/PDK1 cKD parasites cultured in the presence (+Shield-1) or absence of Shield-1 (–Shield-1). Open squares represent data points for individual replicates and the means and SD (error bars) of 3 biological replicate experiments are shown. Differences in SCRs have been compared using a paired 2-tailed Student t test (statistical significance cutoff: p < 0.05). The raw data are available in the source data file (S2 Data). (B) Representative images captured from Giemsa-stained thin blood smears showing the distinct morphology of stage I to V gametocytes cultured under PfPDK1-GFPDD-depleting (–Shield-1) and control (+Shield-1) conditions over 11 days of maturation. Synchronous parasites were split (±Shield-1) as sexual/asexual ring stage parasites 24 hours after the induction of sexual commitment in the preceding IDC. To eliminate asexual parasites, gametocytes were cultured in +SerM supplemented with 50 mM GlcNAc from day 1 to 6 of gametocytogenesis. Scale bar = 5 μm. dgd, day of gametocyte development. (C) Relative ExRs of mature NF54/PDK1 cKD stage V gametocytes (day 14) cultured in presence (+Shield-1) and absence of Shield-1 (–Shield-1). Synchronous parasites were split (±Shield-1) and cultured as described in panel B. Open squares represent data points for individual replicates and the means and SD (error bars) of 3 biological replicate experiments are shown. Differences in ExRs have been compared using an unpaired 2-tailed Student t test (statistical significance cutoff: p < 0.05). The raw data are available in the source data file (S2 Data). ExR, exflagellation rate; IDC, intraerythrocytic developmental cycle; SCR, sexual commitment rate. (TIF) [file pbio.3001483.s010.tif]

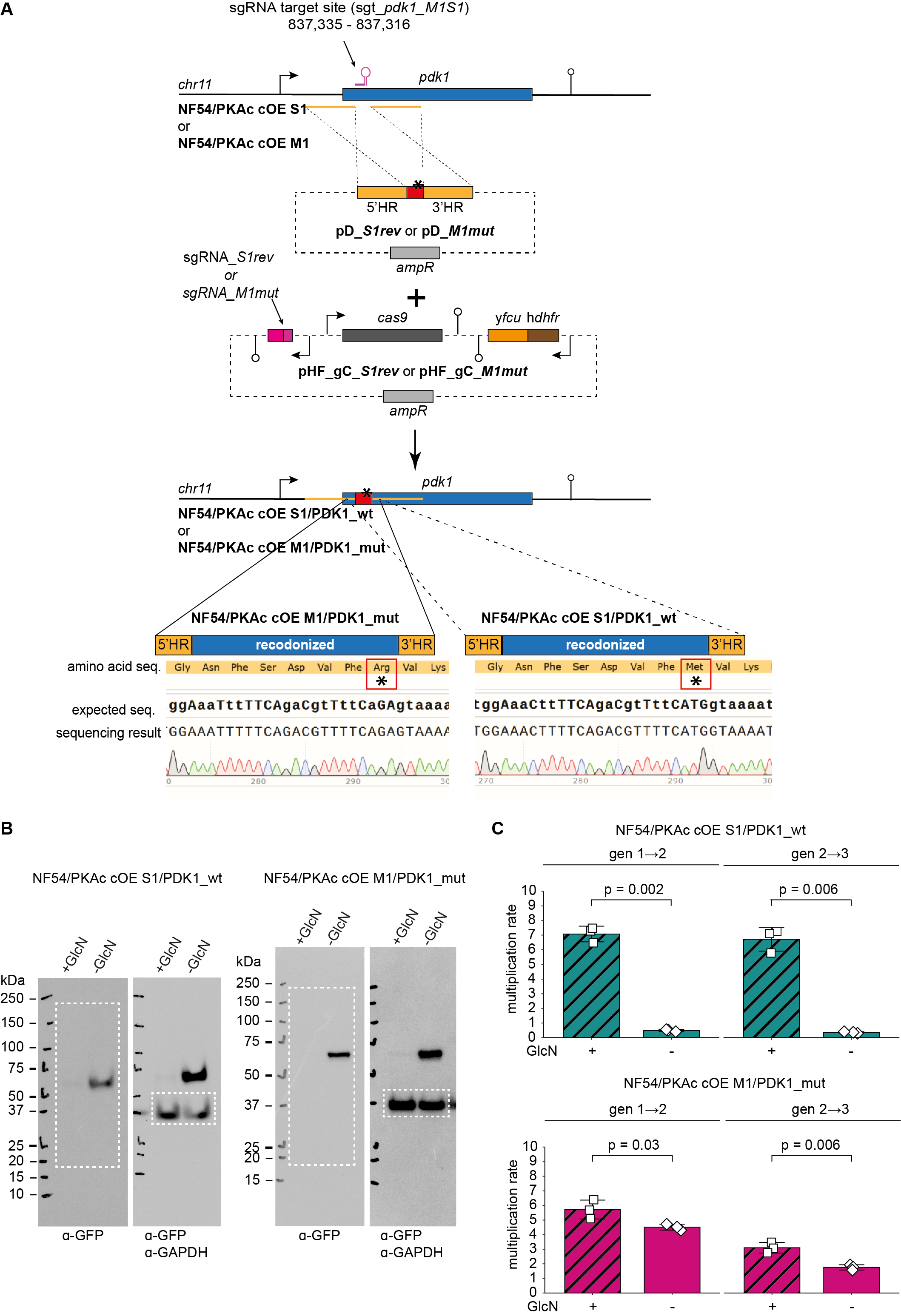

Supplement: S11 Fig — (A) Top: Scheme depicting the pfpdk1 locus of NF54/PKAc cOE S1 or M1 parasites, the donor (pD_S1rev or pD_M1mut) and the suicide (pHF_gC_S1rev or pHF_gC_M1mut) constructs transfected into either NF54/PKAc cOE S1 or NF54/PKAc cOE M1 parasites to generate the NF54/PKAc cOE S1/PDK1_wt and NF54/PKAc cOE M1/PDK1_mut parasite line, respectively, and the edited pfpdk1 locus. Bottom: Sanger sequencing results of modified pfpdk1 genes after targeted mutagenesis in NF54/PKAc cOE S1/PDK1_wt and NF54/PKAc cOE M1/PDK1_mut parasites confirms correct editing. The expected sequences after successful editing, the corresponding amino acid changes and sequencing chromatograms are indicated. The asterisk marks the mutated residues (M51R or R51M). Capital letters highlight the synonymous nucleotide substitutions introduced by CRISPR/Cas-9 editing to destroy the sgRNA target site and to introduce the aspired amino acid change. (B) Full size western blots showing expression of PfPKAc-GFP in NF54/PKAc cOE S1/PDK1_wt (left) and NF54/PKAc cOE M1/PDK1_mut (right) parasites under OE-inducing (–GlcN) and control conditions (+GlcN). Synchronous parasites (0 to 8 hpi) were split (±GlcN) 40 hours before sample collection. Lysates derived from equal numbers of parasites were loaded per lane. The membranes were first probed with α-GFP followed by α-GAPDH control antibodies. MW PfPKAc-GFP = 67.3 kDa, MW PfGAPDH = 36.6 kDa. Dashed lines mark the blot sections shown in Fig 5A and 5B. (C) Parasite multiplication rates of NF54/PKAc cOE S1/PDK1_wt (top) and NF54/PKAc cOE M1/PDK1_mut (bottom) parasites under OE-inducing (–GlcN) and control conditions (+GlcN) over 2 generations. Open squares represent data points for individual replicates and the means and SD (error bars) of 3 biological replicates are shown. Differences in multiplication rates have been compared using a paired 2-tailed Student t test (statistical significance cutoff: p < 0.05). Note that the same data is presented as an increase in parasit [file pbio.3001483.s011.tif]

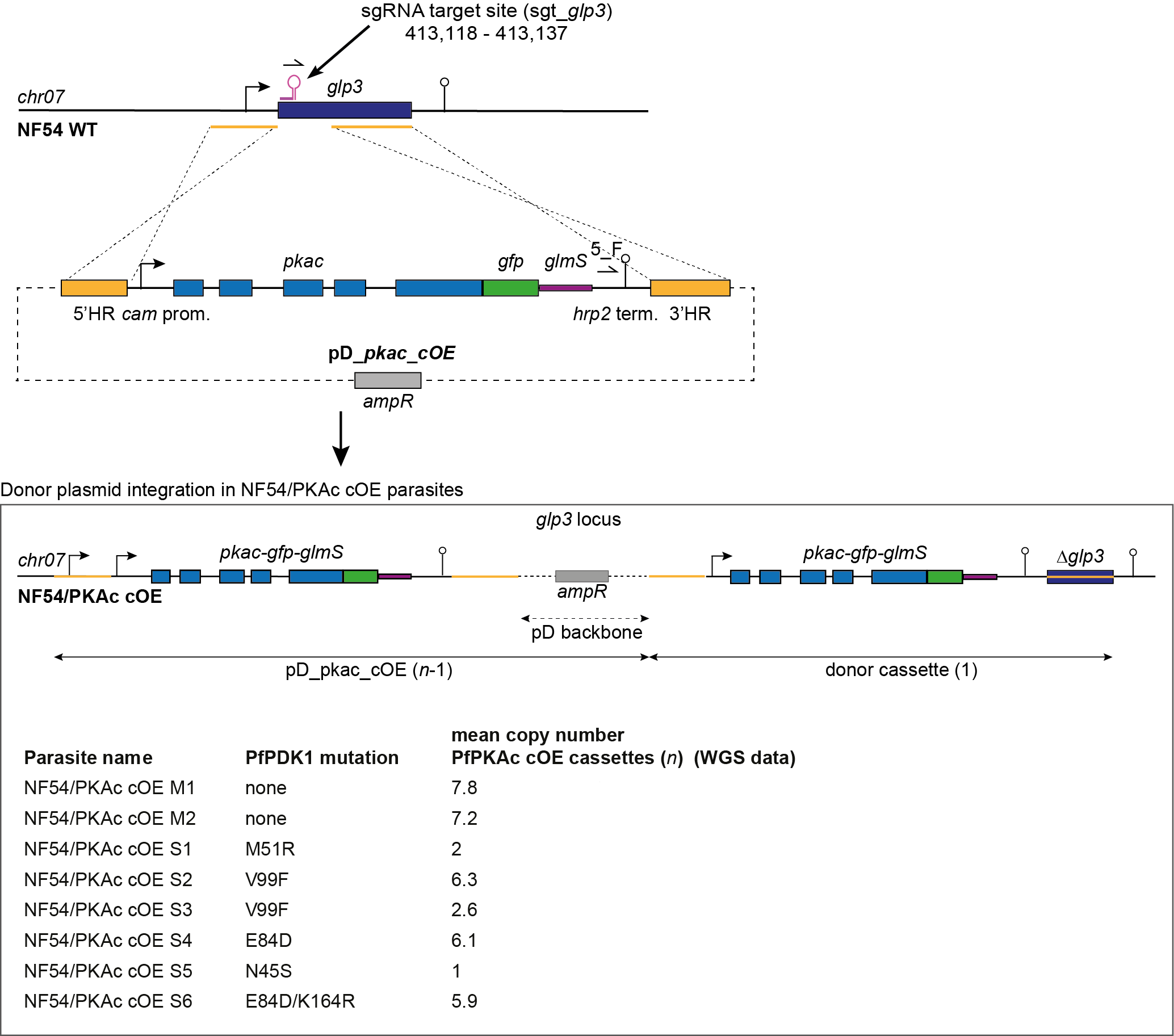

Supplement: S12 Fig — Top: Scheme depicting the WT glp3 target locus and the pD_pkac_cOE donor plasmid used to integrate a PfPKAc cOE cassette into the glp3 locus in NF54 WT parasites to generate the NF54/PKAc cOE parasite line (see also S5 Fig). Bottom: The boxed schematic illustrates the integration of pD_pkac_cOE donor plasmid concatemers into the glp3 locus based on double-crossover recombination of nonadjacent HRs on the concatemer. For reasons of simplicity, the integration of a tandem assembly only is shown. n-1, number of integrated donor plasmids. Estimated mean copy numbers of integrated PfPKAc cOE cassettes (n) are shown for the 2 unselected NF54/PKAc cOE clones (M1, M2) and the 6 independently grown survivor populations (S1-S6), alongside the PfPDK1 mutations identified in the 6 NF54/PKAc cOE survivors (see also Fig 3). Copy numbers of PfPKAc cOE cassettes were calculated from WGS data and the analysis steps are described in the Materials and Methods section. cOE, conditional overexpression; HR, homology region; WGS, whole genome sequencing; WT, wild-type. (TIF) [file pbio.3001483.s012.tif]

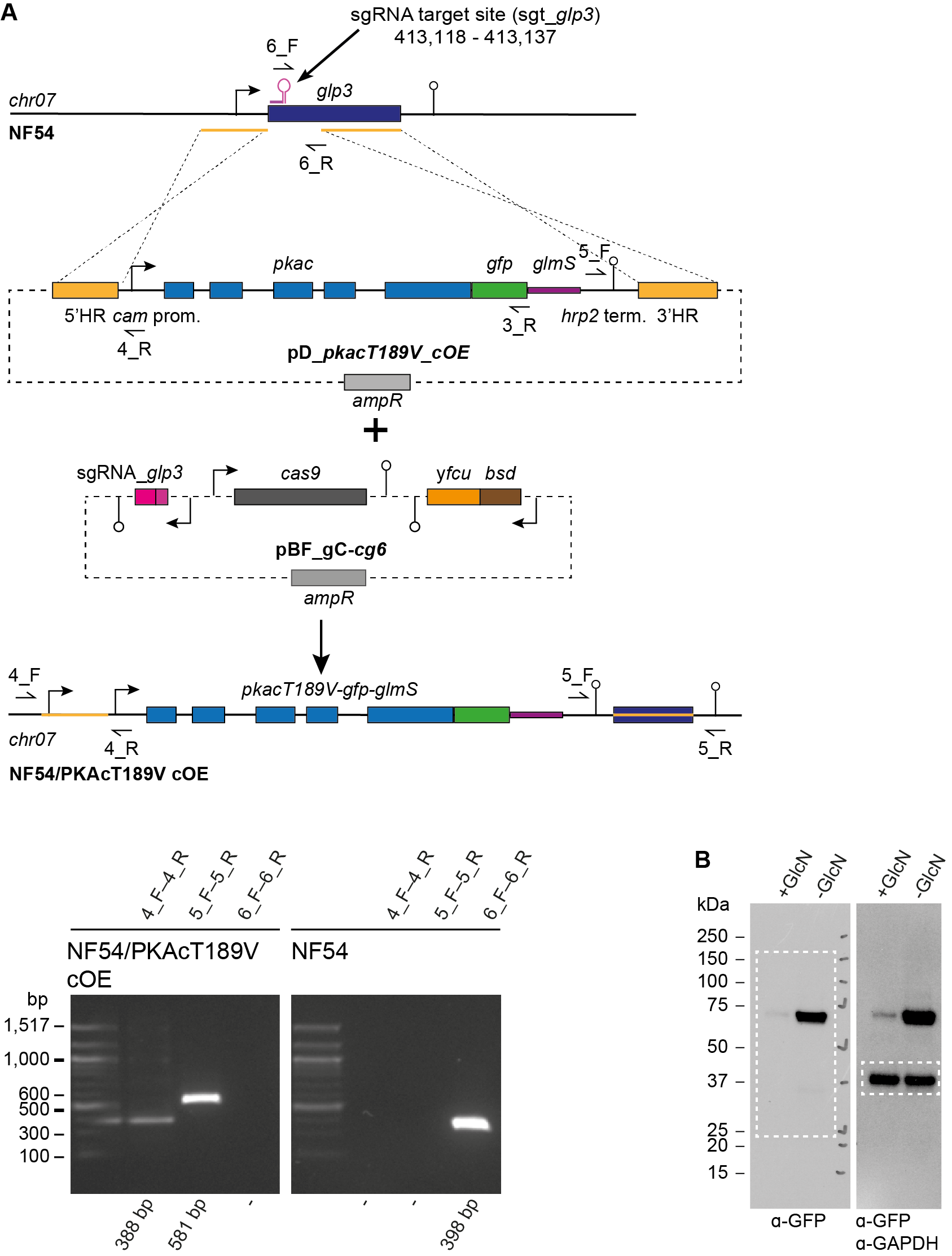

Supplement: S13 Fig — (A) Top: Scheme depicting the WT glp3 target locus, the donor (pD_pkacT189V_cOE) and pBF_gC-cg6 suicide constructs transfected into NF54 WT parasites to generate the NF54/PKAcT189V cOE parasite line and the edited glp3 locus. Primers used for diagnostic PCRs are indicated. Bottom: Results of PCR reactions performed on gDNA of the NF54/PKAcT189V cOE line and NF54 WT control parasites confirm successful insertion of the PfPKAcT189V cOE cassette into the glp3 locus. (B) Full size western blot showing expression of PfPKAcT189V-GFP in NF54/PKAcT189V cOE parasites under OE-inducing (–GlcN) and control conditions (+GlcN). Synchronous parasites (0 to 8 hpi) were split (±GlcN) 40 hours before collection of the samples. Lysates derived from equal numbers of parasites were loaded per lane. The membrane was first probed with α-GFP followed by α-GAPDH control antibodies. MW PfPKAcT189V-GFP = 67.3 kDa, MW PfGAPDH = 36.6 kDa. Dashed lines mark the blot sections shown in Fig 6A. cOE, conditional overexpression; hpi, hours postinvasion; WT, wild-type. (TIF) [file pbio.3001483.s013.tif]

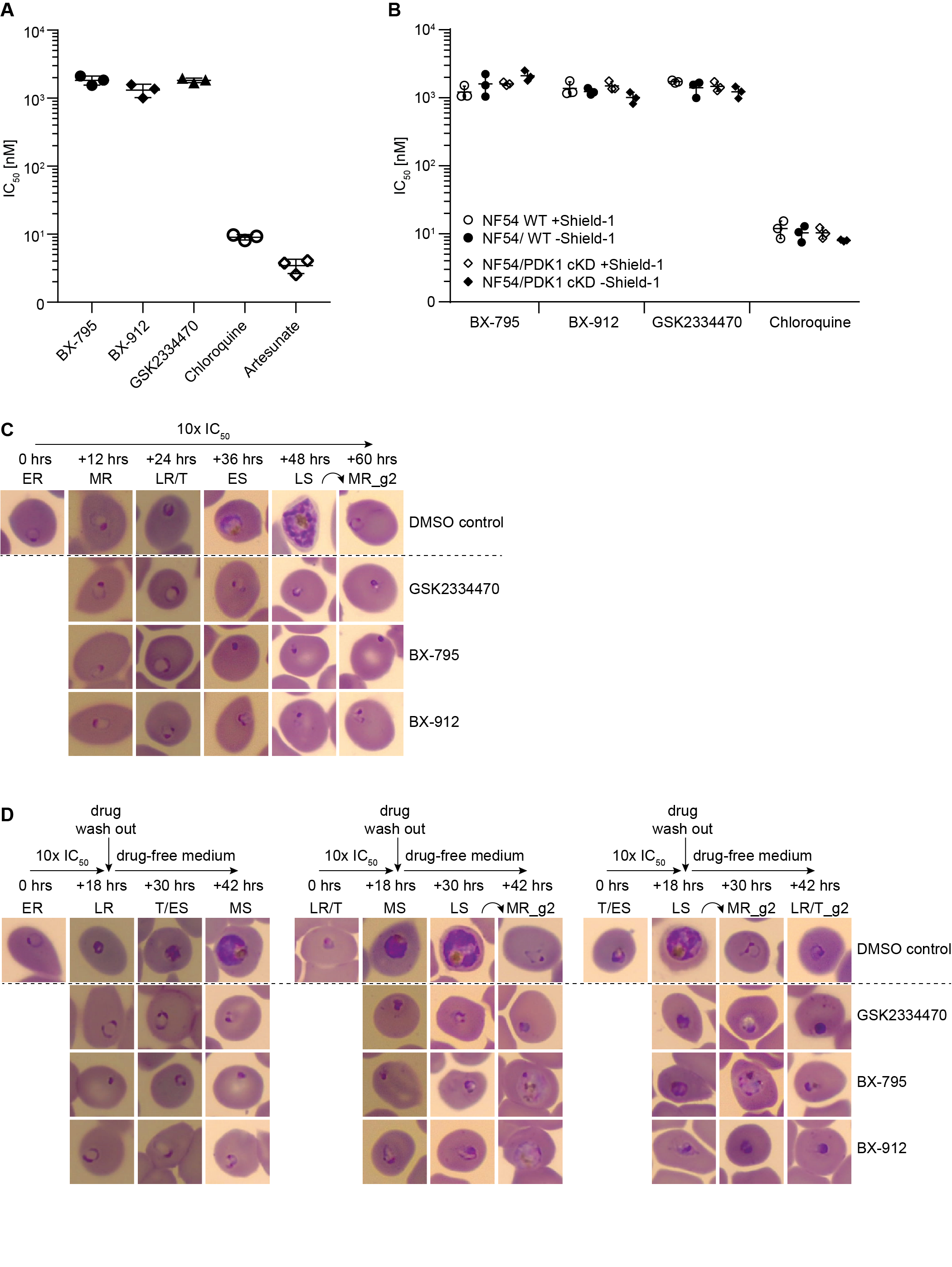

Supplement: S14 Fig — (A, B) IC50 values for the 3 human PDK1 inhibitors BX-795, BX-912, and GSK2334470 and the 2 antimalarial control compounds Chloroquine and Artesunate on the multiplication of NF54 WT parasites (A) and NF54/PDK1 cKD and NF54 WT parasites cultured in the absence (−Shield-1) or presence of Shield-1 (+Shield-1) (B). Symbols represent individual IC50 values calculated from 2 technical replicate dose response assays each. Drug dose response assays were performed in 3 biological replicates, means and SD (error bars) are indicated. The raw data are available in the source data file (S2 Data). (C) Representative images captured from Giemsa-stained thin blood smears of NF54 WT parasites exposed to BX-795, BX-912, and GSK2334470 (10x IC50) and the solvent control (DMSO). Drugs were added to synchronous young ring stage parasites (0 to 4 hpi) and blood smears prepared every 12 hours for 60 hours. The curved arrow marks the time point of merozoite release and invasion into new RBCs in the DMSO control population. (D) Representative images captured from Giemsa-stained thin blood smears of NF54 WT parasites exposed to BX-795, BX-912, and GSK2334470 (10x IC50) and the DMSO solvent control. Drugs were added to synchronous early ring stages (0 to 4 hpi) (left panel), late ring stages/early trophozoites (20 to 24 hpi) (middle panel), or late trophozoites/early schizonts (30 to 34 hpi) (right panel) for 18 hours, followed by drug washout and further culturing in drug-free medium. Blood smears were prepared 18, 30 and, 42 hours after the start of the assay. The curved arrow marks the time point of merozoite release and invasion into new RBCs in the DMSO control population. ER, early ring; ES, early schizont; hpi, hours postinvasion; LR/LR_g2, late rings/late rings generation 2; LR, late ring; LS, late schizont; MR/MR_g2, mid rings/mid rings generation 2; MS, mid schizont; RBC, red blood cell; T, trophozoites; T/T_g2, trophozoites/trophozoites generation 2; WT, wild-type. (TIF) [file pbio.3001483.s014.tif]
