# Supplementary material for: The 3-phosphoinositide–dependent protein kinase 1 is an essential upstream activator of protein kinase A in malaria parasites
Source: PLoS Biol. 2021 Dec 8;19(12):e3001483. doi: 10.1371/journal.pbio.3001483 (PMC8687544; doi:10.1371/journal.pbio.3001483)
Supplement: S1 Table — Names and sequences of oligonucleotides, plasmids and cell lines are indicated. Sequences essential for Gibson assembly reactions (Gibson overhangs) or for T4 DNA ligase-dependent cloning of double-stranded sgRNA-encoding fragments (5′ and 3′ overhangs) are highlighted with capital letters. Italicised letters highlight the annealed sequences (sgRNAs) and colour-highlighted letters represent introduced sequence mutations. sgRNA, single guide RNA. (PDF) [file pbio.3001483.s015.pdf]

Oligo-nucleotide name	Oligonucleotide sequence 5' → 3'	Plasmids	Cell lines
PCRA_F	CTGGCGTAATAGCGAAGAGG	SLI_PKAc_cKD; pD_S1rev; pD_M1mut	NF54/AP2-G-mScarlet/PKAc cKD; NF54/ PKAc cOE S1/PDK1_wt; NF54/PKAc cOE M1/PDK1_mut
PCRA_R	CATTAATGAATCGGCCAACG	SLI_PKAc_cKD; pD_S1rev; pD_M1mut	NF54/AP2-G-mScarlet/PKAc cKD; NF54/ PKAc cOE S1/PDK1_wt; NF54/PKAc cOE M1/PDK1_mut
gfp_F	CGTTGGCCGATTCATTAATGactagtagcataggatccagtggaatgagt	SLI_PKAc_cKD	NF54/AP2-G-mScarlet/PKAc cKD
dd_R	CGCTACCACTTTCCAGTTTTAGAagctcc	SLI_PKAc_cKD	NF54/AP2-G-mScarlet/PKAc cKD
2A_F	TCTAAAACTGGAAAGTGGTAGCGgagaaggaaga	SLI_PKAc_cKD	NF54/AP2-G-mScarlet/PKAc cKD
bsd_R	GAACAAGATTACTCGAGTTAGCCCtcc	SLI_PKAc_cKD	NF54/AP2-G-mScarlet/PKAc cKD
glmS1_F	GGGCTAACTCGAGTAATCTTGTTCttattttctcaatagg	SLI_PKAc_cKD	NF54/AP2-G-mScarlet/PKAc cKD
term_R	CCTCTTCGCTATTACGCCAGgtcgacgaattctagatttaataaatatgttctt	SLI_PKAc_cKD	NF54/AP2-G-mScarlet/PKAc cKD
ydhodh_F	AGAAATATATCATGACAGCCAGtttaactaccaagt	SLI_PKAc_cKD	NF54/AP2-G-mScarlet/PKAc cKD
ydhodh_R	ATATCCTTAATTAATTAAATGCTGTTCaacttcccacg	SLI_PKAc_cKD	NF54/AP2-G-mScarlet/PKAc cKD
pbdt3_F	GAACAGCATTTAATTAATTAAGGATATggcagcttaatgttc	SLI_PKAc_cKD	NF54/AP2-G-mScarlet/PKAc cKD
pbdt3_R	TCGCTATTACGCCAGgtcgacctaccctgaagaagaaaagtcc	SLI_PKAc_cKD	NF54/AP2-G-mScarlet/PKAc cKD
cam1_F	ATTTCTCATTATATATAAGAACATATTTATTAAATgctatccgatcttataaggaaattcc	SLI_PKAc_cKD	NF54/AP2-G-mScarlet/PKAc cKD
cam1_R	CTGGCTGTCATGATATATTTCTattaggtatttattattataaaatataaatcttg	SLI_PKAc_cKD	NF54/AP2-G-mScarlet/PKAc cKD
pka3'_F	CGTTGGCCGATTCATTAATGagctaaaatagtcgagacgagaac	SLI_PKAc_cKD	NF54/AP2-G-mScarlet/PKAc cKD
pka3'_R	TTCTCCTTTACTCATTCCACTGgatccccaatcataaaatggatcattttcatttg	SLI_PKAc_cKD	NF54/AP2-G-mScarlet/PKAc cKD
glp3_F	CTTCTTCAGGGTAGCATGaac	pD_pkac_cOE;	NF54/PKAc cOE
glp3_R	GGATAGCTACATGTTCATAtttatatttatttattc	pD_pkac_cOE;	NF54/PKAc cOE
cam_F	TATGAACATGTAGCTATCCgatcttataaggaaattccc	pD_pkac_cOE;	NF54/PKAc cOE
hrp2_R	CATGCTACCCTGAAGAAGgaattctagatttaataaatatgttc	pD_pkac_cOE;	NF54/PKAc cOE
glmS_F	GCATGGATGAACTATACAAAtaatcttgttcttattttctcaatag	pD_pkac_cOE;	NF54/PKAc cOE
glmS_R	CTCATATACTTCCCTAGATGAGatttttcttcctcctaagattg	pD_pkac_cOE;	NF54/PKAc cOE
clon_F	TAATAAATACCTAATAGAAATATATC*actagtagtggatccagtgga*	pD_pkac_cOE;	NF54/PKAc cOE
clon_R	GAAAAGTTCTTCTCCTTTACTCAT*tccactggatccactactagt*	pD_pkac_cOE;	NF54/PKAc cOE
pka_F	TACCTAATAGAAATATATCACTAGTatgcagtttattaaaaatttgc	pD_pkac_cOE; pD_pkacT189V_cOE	NF54/PKAc cOE
pka_R	CTCCTTTACTCATTCCACTGgatccccaatcataaaatggatcat	pD_pkac_cOE; pD_pkacT189V_cOE	NF54/PKAc cOE
T189V_F	ATGTTTTATGTGGAACTCCAgaatatatc	pD_pkacT189V_cOE	NF54/PKAcT189V cOE
T189V_R	TGGAGTTCCACATAAAACATaagttctcgtctcgac	pD_pkacT189V_cOE	NF54/PKAcT189V cOE
hr1_F	CGTTGGCCGATTCATTAATGgtgtaaaagttaaaaaaacatgc	pD_S1rev; pD_M1mut	NF54/PDK1_S1rev; NF54/PKAc cOE M1/PDK1_mut
rev_F	CTTTTCAGACGTTTTCATGGtaaaattaaaaaatgatccttcaaaaaaatatg	pD_S1rev	NF54/PKAc cOE S1/PDK1_wt
rev_R	CCATGAAAACGTCTGAAAAGtttccagttcctatgtgcatatatatttc	pD_S1rev	NF54/PKAc cOE S1/PDK1_wt
mut_F	TTTTTCAGACGTTTTCAGAGtaaaattaaaaaatgatccttcaaaaaaatatg	pD_M1mut	NF54/PKAc cOE M1/PDK1_mut
mut_R	CTCTGAAAACGTCTGAAAAAtttccagttcctatgtgcatatatatttc	pD_M1mut	NF54/PKAc cOE M1/PDK1_mut
hr2_R	CCTCTTCGCTATTACGCCAGagctattatgtttatttgcatctg	pD_S1rev; pD_M1mut	NF54/PKAc cOE S1/PDK1_wt; NF54/PKAc cOE M1/PDK1_mut
hr1KD_F	CGTTGGCCGATTCATTAATGtagtgacatgtaccgttcc	pD_pdk1-gfpdd	NF54/PDK1 cKD
hr1KD_R	GTTCCCTGGTATCTCTCAAGccacttacttgcttcttccatttc	pD_pdk1-gfpdd	NF54/PDK1 cKD
gfpdd_F	CTTGAGAGATACCAGGGAACtagtggatccagtggaatgagtaaag	pD_pdk1-gfpdd	NF54/PDK1 cKD
gfpdd_R	CTATCATTCTAATTTTAGAAGCTCCACac	pD_pdk1-gfpdd	NF54/PDK1 cKD
hr2KD_F	TGGAGCTTCTAAAATTAGAATGATAGaatataacatatataaataaaaacaatttcttttac	pD_pdk1-gfpdd	NF54/PDK1 cKD
hr2KD_R	CCTCTTCGCTATTACGCCAGgtgtttcacaagaacttaagg	pD_pdk1-gfpdd	NF54/PDK1 cKD
sgRNA_S1rev_F	TATT*gaatttcagtgatgtgttta*	pHF_gC_S1rev	NF54/PKAc cOE S1/PDK1_wt
sgRNA_S1rev_R	AAAC*taaacacatcactgaaattc*	pHF_gC_S1rev	NF54/PKAc cOE S1/PDK1_wt
sgRNA_M1mut_F	TATT*gaatttcagtgatgtgttta*	pHF_gC_M1mut	NF54/PKAc cOE M1/PDK1_mut
sgRNA_M1mut_R	AAAC*taaacacatcactgaaattc*	pHF_gC_M1mut	NF54/PKAc cOE M1/PDK1_mut
sgRNA_pdk1_F	TATT*aaatggttagaacgatatca*	pHF_gC_pdk1-gfpdd	NF54/PDK1 cKD
sgRNA_pdk1_R	AAAC*tgatatcgttctaaccattt*	pHF_gC_pdk1-gfpdd	NF54/PDK1 cKD
