# Supplementary material for: The 3-phosphoinositide–dependent protein kinase 1 is an essential upstream activator of protein kinase A in malaria parasites
Source: PLoS Biol. 2021 Dec 8;19(12):e3001483. doi: 10.1371/journal.pbio.3001483 (PMC8687544; doi:10.1371/journal.pbio.3001483)
Supplement: S2 Table — Names and sequences of oligonucleotides and cell lines are indicated. (PDF) [file pbio.3001483.s016.pdf]

Oligonucleotide name	Oligonucleotide sequence 5' → 3'	Cell lines
1_F	atgcagtttattaaaaatttgc	NF54/AP2-G-mScarlet/PKAc cKD
1_R	gtgtgagttatagttgtattcc	NF54/AP2-G-mScarlet/PKAc cKD
2_F	gcgaggaagcggaagagc	NF54/AP2-G-mScarlet/PKAc cKD
2_R	cattatcaaaacaggcaattg	NF54/AP2-G-mScarlet/PKAc cKD
3_F	ggatcattcaaagatgactc	NF54/AP2-G-mScarlet/PKAc cKD
3_R	gtatgtgaaaacaactaaaacatg	NF54/AP2-G-mScarlet/PKAc cKD
4_F	attatgggaaaataatccttac	NF54/PKAc cOE M1;<br>NF54/PKAc cOE M2<br>NF54/PKAcT189V cOE
4_R	gctcagagattgcatgcaag	NF54/PKAc cOE M1;<br>NF54/PKAc cOE M2<br>NF54/PKAcT189V cOE
5_F	ctttaattttattttggtcatg	NF54/PKAc cOE M1;<br>NF54/PKAc cOE M2<br>NF54/PKAcT189V cOE
5_R	cttttacaatatgaacataaagtac	NF54/PKAc cOE M1;<br>NF54/PKAc cOE M2<br>NF54/PKAcT189V cOE
6_F	gttcatgctcctcaacaaag	NF54/PKAc cOE M1;<br>NF54/PKAc cOE M2<br>NF54/PKAcT189V cOE
6_R	gaacaaatacataagagcgc	NF54/PKAc cOE M1;<br>NF54/PKAc cOE M2<br>NF54/PKAcT189V cOE
7_F	aaaccaggacatgcaaatgttatt	NF54/PDK1 cKD
7_R	tctaatgaattgtccatcatgc	NF54/PDK1 cKD
8_F	ggttatgtacaggaaagaac	NF54/PDK1 cKD
8_R	attcgccattcaggctgc	NF54/PDK1 cKD
9_F	gatcgaaccaagcttatattaac	NF54/PDK1 cKD
10_F	gcgaggaagcggaagagc	NF54/PDK1 cKD
