# Supplementary material for: The 3-phosphoinositide–dependent protein kinase 1 is an essential upstream activator of protein kinase A in malaria parasites
Source: PLoS Biol. 2021 Dec 8;19(12):e3001483. doi: 10.1371/journal.pbio.3001483 (PMC8687544; doi:10.1371/journal.pbio.3001483)
Supplement: S1 Data — The pfpdk1 coding sequences of the NF54/PKAc cOE M1 and M2 clones are identical to the Pf3D7_1121900 reference sequence retrieved from PlasmoDB (www.plasmodb.org) (top row). pfpdk1 coding sequences of the NF54/PKAc cOE survivor populations S1-S6 are shown and deviations from the reference sequence are highlighted in green. NF54/PKAc cOE survivor S6 consists of 2 subpopulations with one carrying the c.252A>T mutation and the other one carrying the c.491A>G mutation (as verified by inspection of the sequencing read pairs). cOE, conditional overexpression; WGS, whole genome sequencing. (PDF) [file pbio.3001483.s017.pdf]

```
                    1                                                                                                  100
PF3D7_1121900       ATGAAGAAAGGATTTTTGTTGAATAAGAATATATATGATATTGAAGAAAATGTAATAAATGTTGAAAAAAATAACACAAATAAAAAATATTGTAAGGATG
S1_c.152T>G         ATGAAGAAAGGATTTTTGTTGAATAAGAATATATATGATATTGAAGAAAATGTAATAAATGTTGAAAAAAATAACACAAATAAAAAATATTGTAAGGATG
S2_c.295G>T         ATGAAGAAAGGATTTTTGTTGAATAAGAATATATATGATATTGAAGAAAATGTAATAAATGTTGAAAAAAATAACACAAATAAAAAATATTGTAAGGATG
S3_c.295G>T         ATGAAGAAAGGATTTTTGTTGAATAAGAATATATATGATATTGAAGAAAATGTAATAAATGTTGAAAAAAATAACACAAATAAAAAATATTGTAAGGATG
S4_c.252A>T         ATGAAGAAAGGATTTTTGTTGAATAAGAATATATATGATATTGAAGAAAATGTAATAAATGTTGAAAAAAATAACACAAATAAAAAATATTGTAAGGATG
S5_c.134A>G         ATGAAGAAAGGATTTTTGTTGAATAAGAATATATATGATATTGAAGAAAATGTAATAAATGTTGAAAAAAATAACACAAATAAAAAATATTGTAAGGATG
S6_c.252A>T         ATGAAGAAAGGATTTTTGTTGAATAAGAATATATATGATATTGAAGAAAATGTAATAAATGTTGAAAAAAATAACACAAATAAAAAATATTGTAAGGATG
S6_c.491A>G         ATGAAGAAAGGATTTTTGTTGAATAAGAATATATATGATATTGAAGAAAATGTAATAAATGTTGAAAAAAATAACACAAATAAAAAATATTGTAAGGATG

                    101                                                                                                200
PF3D7_1121900       ATTTTGAAATATATATGCACATAGGAACTGGGAATTTCAGTGATGTGTTTATGGTAAAATTAAAAAATGATCCTTCAAAAAAATATGCCTTAAAAATATT
S1_c.152T>G         ATTTTGAAATATATATGCACATAGGAACTGGGAATTTCAGTGATGTGTTTAGGGTAAAATTAAAAAATGATCCTTCAAAAAAATATGCCTTAAAAATATT
S2_c.295G>T         ATTTTGAAATATATATGCACATAGGAACTGGGAATTTCAGTGATGTGTTTATGGTAAAATTAAAAAATGATCCTTCAAAAAAATATGCCTTAAAAATATT
S3_c.295G>T         ATTTTGAAATATATATGCACATAGGAACTGGGAATTTCAGTGATGTGTTTATGGTAAAATTAAAAAATGATCCTTCAAAAAAATATGCCTTAAAAATATT
S4_c.252A>T         ATTTTGAAATATATATGCACATAGGAACTGGGAATTTCAGTGATGTGTTTATGGTAAAATTAAAAAATGATCCTTCAAAAAAATATGCCTTAAAAATATT
S5_c.134A>G         ATTTTGAAATATATATGCACATAGGAACTGGGAGTTTCAGTGATGTGTTTATGGTAAAATTAAAAAATGATCCTTCAAAAAAATATGCCTTAAAAATATT
S6_c.252A>T         ATTTTGAAATATATATGCACATAGGAACTGGGAATTTCAGTGATGTGTTTATGGTAAAATTAAAAAATGATCCTTCAAAAAAATATGCCTTAAAAATATT
S6_c.491A>G         ATTTTGAAATATATATGCACATAGGAACTGGGAATTTCAGTGATGTGTTTATGGTAAAATTAAAAAATGATCCTTCAAAAAAATATGCCTTAAAAATATT

                    201                                                                                                300
PF3D7_1121900       CAAAAAAGAAAAAGTGAATAAAATGAATAAAGTAAATGATGTGTTAACAGAAAAAAACGTTATGTCAAAATTAAATACCCCAGGACATGCAAATGTTATT
S1_c.152T>G         CAAAAAAGAAAAAGTGAATAAAATGAATAAAGTAAATGATGTGTTAACAGAAAAAAACGTTATGTCAAAATTAAATACCCCAGGACATGCAAATGTTATT
S2_c.295G>T         CAAAAAAGAAAAAGTGAATAAAATGAATAAAGTAAATGATGTGTTAACAGAAAAAAACGTTATGTCAAAATTAAATACCCCAGGACATGCAAATTTTATT
S3_c.295G>T         CAAAAAAGAAAAAGTGAATAAAATGAATAAAGTAAATGATGTGTTAACAGAAAAAAACGTTATGTCAAAATTAAATACCCCAGGACATGCAAATTTTATT
S4_c.252A>T         CAAAAAAGAAAAAGTGAATAAAATGAATAAAGTAAATGATGTGTTAACAGATAAAAACGTTATGTCAAAATTAAATACCCCAGGACATGCAAATGTTATT
S5_c.134A>G         CAAAAAAGAAAAAGTGAATAAAATGAATAAAGTAAATGATGTGTTAACAGAAAAAAACGTTATGTCAAAATTAAATACCCCAGGACATGCAAATGTTATT
S6_c.252A>T         CAAAAAAGAAAAAGTGAATAAAATGAATAAAGTAAATGATGTGTTAACAGATAAAAACGTTATGTCAAAATTAAATACCCCAGGACATGCAAATGTTATT
S6_c.491A>G         CAAAAAAGAAAAAGTGAATAAAATGAATAAAGTAAATGATGTGTTAACAGAAAAAAACGTTATGTCAAAATTAAATACCCCAGGACATGCAAATGTTATT

                    301                                                                                                400
PF3D7_1121900       AAATTAATAGAAACATTTAAAGACAAAGAAAATGTTTATTTATTATATGAATATGCTGATTATGATTTATGGGAATTTTTAAAAATTCGTAGTGTTGGTG
S1_c.152T>G         AAATTAATAGAAACATTTAAAGACAAAGAAAATGTTTATTTATTATATGAATATGCTGATTATGATTTATGGGAATTTTTAAAAATTCGTAGTGTTGGTG
S2_c.295G>T         AAATTAATAGAAACATTTAAAGACAAAGAAAATGTTTATTTATTATATGAATATGCTGATTATGATTTATGGGAATTTTTAAAAATTCGTAGTGTTGGTG
S3_c.295G>T         AAATTAATAGAAACATTTAAAGACAAAGAAAATGTTTATTTATTATATGAATATGCTGATTATGATTTATGGGAATTTTTAAAAATTCGTAGTGTTGGTG
S4_c.252A>T         AAATTAATAGAAACATTTAAAGACAAAGAAAATGTTTATTTATTATATGAATATGCTGATTATGATTTATGGGAATTTTTAAAAATTCGTAGTGTTGGTG
S5_c.134A>G         AAATTAATAGAAACATTTAAAGACAAAGAAAATGTTTATTTATTATATGAATATGCTGATTATGATTTATGGGAATTTTTAAAAATTCGTAGTGTTGGTG
S6_c.252A>T         AAATTAATAGAAACATTTAAAGACAAAGAAAATGTTTATTTATTATATGAATATGCTGATTATGATTTATGGGAATTTTTAAAAATTCGTAGTGTTGGTG
S6_c.491A>G         AAATTAATAGAAACATTTAAAGACAAAGAAAATGTTTATTTATTATATGAATATGCTGATTATGATTTATGGGAATTTTTAAAAATTCGTAGTGTTGGTG

                    401                                                                                                500
PF3D7_1121900       TTAATGAAAAAATTACATTTAATATAATACTTCAAATGGTACATGCATTAATATATATACATAACAAAAATATTATTCACAGAGACTTGAAATGTGAAAA
S1_c.152T>G         TTAATGAAAAAATTACATTTAATATAATACTTCAAATGGTACATGCATTAATATATATACATAACAAAAATATTATTCACAGAGACTTGAAATGTGAAAA
S2_c.295G>T         TTAATGAAAAAATTACATTTAATATAATACTTCAAATGGTACATGCATTAATATATATACATAACAAAAATATTATTCACAGAGACTTGAAATGTGAAAA
S3_c.295G>T         TTAATGAAAAAATTACATTTAATATAATACTTCAAATGGTACATGCATTAATATATATACATAACAAAAATATTATTCACAGAGACTTGAAATGTGAAAA
S4_c.252A>T         TTAATGAAAAAATTACATTTAATATAATACTTCAAATGGTACATGCATTAATATATATACATAACAAAAATATTATTCACAGAGACTTGAAATGTGAAAA
S5_c.134A>G         TTAATGAAAAAATTACATTTAATATAATACTTCAAATGGTACATGCATTAATATATATACATAACAAAAATATTATTCACAGAGACTTGAAATGTGAAAA
S6_c.252A>T         TTAATGAAAAAATTACATTTAATATAATACTTCAAATGGTACATGCATTAATATATATACATAACAAAAATATTATTCACAGAGACTTGAAATGTGAAAA
S6_c.491A>G         TTAATGAAAAAATTACATTTAATATAATACTTCAAATGGTACATGCATTAATATATATACATAACAAAAATATTATTCACAGAGACTTGAGATGTGAAAA

                    501                                                                                                600
PF3D7_1121900       TTTTCTTATAAATAAAGATGGAACCATAAAAATGATTGATTTTGGTAGTTCGAAAGATTTAGATAACATAAGCATCAAAACAAATAATGAGGAAGATACA
S1_c.152T>G         TTTTCTTATAAATAAAGATGGAACCATAAAAATGATTGATTTTGGTAGTTCGAAAGATTTAGATAACATAAGCATCAAAACAAATAATGAGGAAGATACA
S2_c.295G>T         TTTTCTTATAAATAAAGATGGAACCATAAAAATGATTGATTTTGGTAGTTCGAAAGATTTAGATAACATAAGCATCAAAACAAATAATGAGGAAGATACA
S3_c.295G>T         TTTTCTTATAAATAAAGATGGAACCATAAAAATGATTGATTTTGGTAGTTCGAAAGATTTAGATAACATAAGCATCAAAACAAATAATGAGGAAGATACA
S4_c.252A>T         TTTTCTTATAAATAAAGATGGAACCATAAAAATGATTGATTTTGGTAGTTCGAAAGATTTAGATAACATAAGCATCAAAACAAATAATGAGGAAGATACA
S5_c.134A>G         TTTTCTTATAAATAAAGATGGAACCATAAAAATGATTGATTTTGGTAGTTCGAAAGATTTAGATAACATAAGCATCAAAACAAATAATGAGGAAGATACA
S6_c.252A>T         TTTTCTTATAAATAAAGATGGAACCATAAAAATGATTGATTTTGGTAGTTCGAAAGATTTAGATAACATAAGCATCAAAACAAATAATGAGGAAGATACA
S6_c.491A>G         TTTTCTTATAAATAAAGATGGAACCATAAAAATGATTGATTTTGGTAGTTCGAAAGATTTAGATAACATAAGCATCAAAACAAATAATGAGGAAGATACA

                    601                                                                                                700
PF3D7_1121900       ATAAATCATGAAGAATTAAGTAAATTTGTATTAAAAAAAAATAATAACAATAATTCAAACGAAGATTTAAAAAATGCAAATGAATTTAAAAATAATGATA
S1_c.152T>G         ATAAATCATGAAGAATTAAGTAAATTTGTATTAAAAAAAAATAATAACAATAATTCAAACGAAGATTTAAAAAATGCAAATGAATTTAAAAATAATGATA
S2_c.295G>T         ATAAATCATGAAGAATTAAGTAAATTTGTATTAAAAAAAAATAATAACAATAATTCAAACGAAGATTTAAAAAATGCAAATGAATTTAAAAATAATGATA
S3_c.295G>T         ATAAATCATGAAGAATTAAGTAAATTTGTATTAAAAAAAAATAATAACAATAATTCAAACGAAGATTTAAAAAATGCAAATGAATTTAAAAATAATGATA
S4_c.252A>T         ATAAATCATGAAGAATTAAGTAAATTTGTATTAAAAAAAAATAATAACAATAATTCAAACGAAGATTTAAAAAATGCAAATGAATTTAAAAATAATGATA
S5_c.134A>G         ATAAATCATGAAGAATTAAGTAAATTTGTATTAAAAAAAAATAATAACAATAATTCAAACGAAGATTTAAAAAATGCAAATGAATTTAAAAATAATGATA
S6_c.252A>T         ATAAATCATGAAGAATTAAGTAAATTTGTATTAAAAAAAAATAATAACAATAATTCAAACGAAGATTTAAAAAATGCAAATGAATTTAAAAATAATGATA
S6_c.491A>G         ATAAATCATGAAGAATTAAGTAAATTTGTATTAAAAAAAAATAATAACAATAATTCAAACGAAGATTTAAAAAATGCAAATGAATTTAAAAATAATGATA

                    701                                                                                                800
PF3D7_1121900       GTTTAAATGGCGAAGAAATTAATAATAATGATTTAAATAATACTAATTTTCAAAAAAGTGATAAAAATATAAAAGATCAAAATTCAAACAAATGCGTTCT
S1_c.152T>G         GTTTAAATGGCGAAGAAATTAATAATAATGATTTAAATAATACTAATTTTCAAAAAAGTGATAAAAATATAAAAGATCAAAATTCAAACAAATGCGTTCT
S2_c.295G>T         GTTTAAATGGCGAAGAAATTAATAATAATGATTTAAATAATACTAATTTTCAAAAAAGTGATAAAAATATAAAAGATCAAAATTCAAACAAATGCGTTCT
S3_c.295G>T         GTTTAAATGGCGAAGAAATTAATAATAATGATTTAAATAATACTAATTTTCAAAAAAGTGATAAAAATATAAAAGATCAAAATTCAAACAAATGCGTTCT
S4_c.252A>T         GTTTAAATGGCGAAGAAATTAATAATAATGATTTAAATAATACTAATTTTCAAAAAAGTGATAAAAATATAAAAGATCAAAATTCAAACAAATGCGTTCT
S5_c.134A>G         GTTTAAATGGCGAAGAAATTAATAATAATGATTTAAATAATACTAATTTTCAAAAAAGTGATAAAAATATAAAAGATCAAAATTCAAACAAATGCGTTCT
S6_c.252A>T         GTTTAAATGGCGAAGAAATTAATAATAATGATTTAAATAATACTAATTTTCAAAAAAGTGATAAAAATATAAAAGATCAAAATTCAAACAAATGCGTTCT
S6_c.491A>G         GTTTAAATGGCGAAGAAATTAATAATAATGATTTAAATAATACTAATTTTCAAAAAAGTGATAAAAATATAAAAGATCAAAATTCAAACAAATGCGTTCT

                    801                                                                                                900
PF3D7_1121900       AGAAACACTTAAGAATAATGAAAGCTATGAGTTTAATAATCAAATTTTGAGTTCTTTCAAAAGTAACGATTATGCAGCAGATGCAAATAAACATAATAGC
S1_c.152T>G         AGAAACACTTAAGAATAATGAAAGCTATGAGTTTAATAATCAAATTTTGAGTTCTTTCAAAAGTAACGATTATGCAGCAGATGCAAATAAACATAATAGC
S2_c.295G>T         AGAAACACTTAAGAATAATGAAAGCTATGAGTTTAATAATCAAATTTTGAGTTCTTTCAAAAGTAACGATTATGCAGCAGATGCAAATAAACATAATAGC
S3_c.295G>T         AGAAACACTTAAGAATAATGAAAGCTATGAGTTTAATAATCAAATTTTGAGTTCTTTCAAAAGTAACGATTATGCAGCAGATGCAAATAAACATAATAGC
S4_c.252A>T         AGAAACACTTAAGAATAATGAAAGCTATGAGTTTAATAATCAAATTTTGAGTTCTTTCAAAAGTAACGATTATGCAGCAGATGCAAATAAACATAATAGC
S5_c.134A>G         AGAAACACTTAAGAATAATGAAAGCTATGAGTTTAATAATCAAATTTTGAGTTCTTTCAAAAGTAACGATTATGCAGCAGATGCAAATAAACATAATAGC
S6_c.252A>T         AGAAACACTTAAGAATAATGAAAGCTATGAGTTTAATAATCAAATTTTGAGTTCTTTCAAAAGTAACGATTATGCAGCAGATGCAAATAAACATAATAGC
S6_c.491A>G         AGAAACACTTAAGAATAATGAAAGCTATGAGTTTAATAATCAAATTTTGAGTTCTTTCAAAAGTAACGATTATGCAGCAGATGCAAATAAACATAATAGC
```

```
                    901                                                                                                1000
PF3D7_1121900       TATAAAAAAAAAAAAACATTCGAAAACTATGTTGGTTCTCCTAATTTCATTCCCCCTGAAGCATTAATAAATAAATGCAGTGGTAAGGCACGAGATTTTT
S1_c.152T>G         TATAAAAAAAAAAAAACATTCGAAAACTATGTTGGTTCTCCTAATTTCATTCCCCCTGAAGCATTAATAAATAAATGCAGTGGTAAGGCACGAGATTTTT
S2_c.295G>T         TATAAAAAAAAAAAAACATTCGAAAACTATGTTGGTTCTCCTAATTTCATTCCCCCTGAAGCATTAATAAATAAATGCAGTGGTAAGGCACGAGATTTTT
S3_c.295G>T         TATAAAAAAAAAAAAACATTCGAAAACTATGTTGGTTCTCCTAATTTCATTCCCCCTGAAGCATTAATAAATAAATGCAGTGGTAAGGCACGAGATTTTT
S4_c.252A>T         TATAAAAAAAAAAAAACATTCGAAAACTATGTTGGTTCTCCTAATTTCATTCCCCCTGAAGCATTAATAAATAAATGCAGTGGTAAGGCACGAGATTTTT
S5_c.134A>G         TATAAAAAAAAAAAAACATTCGAAAACTATGTTGGTTCTCCTAATTTCATTCCCCCTGAAGCATTAATAAATAAATGCAGTGGTAAGGCACGAGATTTTT
S6_c.252A>T         TATAAAAAAAAAAAAACATTCGAAAACTATGTTGGTTCTCCTAATTTCATTCCCCCTGAAGCATTAATAAATAAATGCAGTGGTAAGGCACGAGATTTTT
S6_c.491A>G         TATAAAAAAAAAAAAACATTCGAAAACTATGTTGGTTCTCCTAATTTCATTCCCCCTGAAGCATTAATAAATAAATGCAGTGGTAAGGCACGAGATTTTT

                    1001                                                                                               1100
PF3D7_1121900       GGAGTCTTGGGTGTACTATTTACCAACTAGTGACATGTACCGTTCCATTTGATGGTTCAACAGAATGGTTCATCTATAACAAAATAAAAAGAAGGGAATT
S1_c.152T>G         GGAGTCTTGGGTGTACTATTTACCAACTAGTGACATGTACCGTTCCATTTGATGGTTCAACAGAATGGTTCATCTATAACAAAATAAAAAGAAGGGAATT
S2_c.295G>T         GGAGTCTTGGGTGTACTATTTACCAACTAGTGACATGTACCGTTCCATTTGATGGTTCAACAGAATGGTTCATCTATAACAAAATAAAAAGAAGGGAATT
S3_c.295G>T         GGAGTCTTGGGTGTACTATTTACCAACTAGTGACATGTACCGTTCCATTTGATGGTTCAACAGAATGGTTCATCTATAACAAAATAAAAAGAAGGGAATT
S4_c.252A>T         GGAGTCTTGGGTGTACTATTTACCAACTAGTGACATGTACCGTTCCATTTGATGGTTCAACAGAATGGTTCATCTATAACAAAATAAAAAGAAGGGAATT
S5_c.134A>G         GGAGTCTTGGGTGTACTATTTACCAACTAGTGACATGTACCGTTCCATTTGATGGTTCAACAGAATGGTTCATCTATAACAAAATAAAAAGAAGGGAATT
S6_c.252A>T         GGAGTCTTGGGTGTACTATTTACCAACTAGTGACATGTACCGTTCCATTTGATGGTTCAACAGAATGGTTCATCTATAACAAAATAAAAAGAAGGGAATT
S6_c.491A>G         GGAGTCTTGGGTGTACTATTTACCAACTAGTGACATGTACCGTTCCATTTGATGGTTCAACAGAATGGTTCATCTATAACAAAATAAAAAGAAGGGAATT

                    1101                                                                                               1200
PF3D7_1121900       AAAATATCCATCCATAATACCTTCTGAATTAATAGACTTAATAGAAAAGTTAACAACTATGAATCCAGAAGAAAGATTAGGGTTTAACGGAGGATGTGAG
S1_c.152T>G         AAAATATCCATCCATAATACCTTCTGAATTAATAGACTTAATAGAAAAGTTAACAACTATGAATCCAGAAGAAAGATTAGGGTTTAACGGAGGATGTGAG
S2_c.295G>T         AAAATATCCATCCATAATACCTTCTGAATTAATAGACTTAATAGAAAAGTTAACAACTATGAATCCAGAAGAAAGATTAGGGTTTAACGGAGGATGTGAG
S3_c.295G>T         AAAATATCCATCCATAATACCTTCTGAATTAATAGACTTAATAGAAAAGTTAACAACTATGAATCCAGAAGAAAGATTAGGGTTTAACGGAGGATGTGAG
S4_c.252A>T         AAAATATCCATCCATAATACCTTCTGAATTAATAGACTTAATAGAAAAGTTAACAACTATGAATCCAGAAGAAAGATTAGGGTTTAACGGAGGATGTGAG
S5_c.134A>G         AAAATATCCATCCATAATACCTTCTGAATTAATAGACTTAATAGAAAAGTTAACAACTATGAATCCAGAAGAAAGATTAGGGTTTAACGGAGGATGTGAG
S6_c.252A>T         AAAATATCCATCCATAATACCTTCTGAATTAATAGACTTAATAGAAAAGTTAACAACTATGAATCCAGAAGAAAGATTAGGGTTTAACGGAGGATGTGAG
S6_c.491A>G         AAAATATCCATCCATAATACCTTCTGAATTAATAGACTTAATAGAAAAGTTAACAACTATGAATCCAGAAGAAAGATTAGGGTTTAACGGAGGATGTGAG

                    1201                                                                                               1300
PF3D7_1121900       GAAATCTTGGAACATTTATATTTTCAAAAATATAATTATAATAAACTTAATTTCATCTTACCAGAAGTTTCCGAGTTAGAAAAACTGTATACAACTATTA
S1_c.152T>G         GAAATCTTGGAACATTTATATTTTCAAAAATATAATTATAATAAACTTAATTTCATCTTACCAGAAGTTTCCGAGTTAGAAAAACTGTATACAACTATTA
S2_c.295G>T         GAAATCTTGGAACATTTATATTTTCAAAAATATAATTATAATAAACTTAATTTCATCTTACCAGAAGTTTCCGAGTTAGAAAAACTGTATACAACTATTA
S3_c.295G>T         GAAATCTTGGAACATTTATATTTTCAAAAATATAATTATAATAAACTTAATTTCATCTTACCAGAAGTTTCCGAGTTAGAAAAACTGTATACAACTATTA
S4_c.252A>T         GAAATCTTGGAACATTTATATTTTCAAAAATATAATTATAATAAACTTAATTTCATCTTACCAGAAGTTTCCGAGTTAGAAAAACTGTATACAACTATTA
S5_c.134A>G         GAAATCTTGGAACATTTATATTTTCAAAAATATAATTATAATAAACTTAATTTCATCTTACCAGAAGTTTCCGAGTTAGAAAAACTGTATACAACTATTA
S6_c.252A>T         GAAATCTTGGAACATTTATATTTTCAAAAATATAATTATAATAAACTTAATTTCATCTTACCAGAAGTTTCCGAGTTAGAAAAACTGTATACAACTATTA
S6_c.491A>G         GAAATCTTGGAACATTTATATTTTCAAAAATATAATTATAATAAACTTAATTTCATCTTACCAGAAGTTTCCGAGTTAGAAAAACTGTATACAACTATTA

                    1301                                                                                               1400
PF3D7_1121900       TAAATAAGTATCATATATATATCAATGAAAAGAGGAAATTAAGACAAAATAATAATTCAACTGAAGAAAATATTAATAATGTAGAAGTACTGAAAAAAAA
S1_c.152T>G         TAAATAAGTATCATATATATATCAATGAAAAGAGGAAATTAAGACAAAATAATAATTCAACTGAAGAAAATATTAATAATGTAGAAGTACTGAAAAAAAA
S2_c.295G>T         TAAATAAGTATCATATATATATCAATGAAAAGAGGAAATTAAGACAAAATAATAATTCAACTGAAGAAAATATTAATAATGTAGAAGTACTGAAAAAAAA
S3_c.295G>T         TAAATAAGTATCATATATATATCAATGAAAAGAGGAAATTAAGACAAAATAATAATTCAACTGAAGAAAATATTAATAATGTAGAAGTACTGAAAAAAAA
S4_c.252A>T         TAAATAAGTATCATATATATATCAATGAAAAGAGGAAATTAAGACAAAATAATAATTCAACTGAAGAAAATATTAATAATGTAGAAGTACTGAAAAAAAA
S5_c.134A>G         TAAATAAGTATCATATATATATCAATGAAAAGAGGAAATTAAGACAAAATAATAATTCAACTGAAGAAAATATTAATAATGTAGAAGTACTGAAAAAAAA
S6_c.252A>T         TAAATAAGTATCATATATATATCAATGAAAAGAGGAAATTAAGACAAAATAATAATTCAACTGAAGAAAATATTAATAATGTAGAAGTACTGAAAAAAAA
S6_c.491A>G         TAAATAAGTATCATATATATATCAATGAAAAGAGGAAATTAAGACAAAATAATAATTCAACTGAAGAAAATATTAATAATGTAGAAGTACTGAAAAAAAA

                    1401                                                                                               1500
PF3D7_1121900       TTTGTTAAACTTAATTAATTCAGATACATTAGTTTGTGCTGAGGAGGAATACGAATCCATAACTTTAAAGAAGAAAATTTTTAAAAGTATTAATTTTATG
S1_c.152T>G         TTTGTTAAACTTAATTAATTCAGATACATTAGTTTGTGCTGAGGAGGAATACGAATCCATAACTTTAAAGAAGAAAATTTTTAAAAGTATTAATTTTATG
S2_c.295G>T         TTTGTTAAACTTAATTAATTCAGATACATTAGTTTGTGCTGAGGAGGAATACGAATCCATAACTTTAAAGAAGAAAATTTTTAAAAGTATTAATTTTATG
S3_c.295G>T         TTTGTTAAACTTAATTAATTCAGATACATTAGTTTGTGCTGAGGAGGAATACGAATCCATAACTTTAAAGAAGAAAATTTTTAAAAGTATTAATTTTATG
S4_c.252A>T         TTTGTTAAACTTAATTAATTCAGATACATTAGTTTGTGCTGAGGAGGAATACGAATCCATAACTTTAAAGAAGAAAATTTTTAAAAGTATTAATTTTATG
S5_c.134A>G         TTTGTTAAACTTAATTAATTCAGATACATTAGTTTGTGCTGAGGAGGAATACGAATCCATAACTTTAAAGAAGAAAATTTTTAAAAGTATTAATTTTATG
S6_c.252A>T         TTTGTTAAACTTAATTAATTCAGATACATTAGTTTGTGCTGAGGAGGAATACGAATCCATAACTTTAAAGAAGAAAATTTTTAAAAGTATTAATTTTATG
S6_c.491A>G         TTTGTTAAACTTAATTAATTCAGATACATTAGTTTGTGCTGAGGAGGAATACGAATCCATAACTTTAAAGAAGAAAATTTTTAAAAGTATTAATTTTATG

                    1501                                                                          1578
PF3D7_1121900       CTTGAAGAATTTGATAAACAGGAAATAAAAGAAATGGAAGAAGCAAGTAAATGGTTAGAACGATATCAAGGTACATAG
S1_c.152T>G         CTTGAAGAATTTGATAAACAGGAAATAAAAGAAATGGAAGAAGCAAGTAAATGGTTAGAACGATATCAAGGTACATAG
S2_c.295G>T         CTTGAAGAATTTGATAAACAGGAAATAAAAGAAATGGAAGAAGCAAGTAAATGGTTAGAACGATATCAAGGTACATAG
S3_c.295G>T         CTTGAAGAATTTGATAAACAGGAAATAAAAGAAATGGAAGAAGCAAGTAAATGGTTAGAACGATATCAAGGTACATAG
S4_c.252A>T         CTTGAAGAATTTGATAAACAGGAAATAAAAGAAATGGAAGAAGCAAGTAAATGGTTAGAACGATATCAAGGTACATAG
S5_c.134A>G         CTTGAAGAATTTGATAAACAGGAAATAAAAGAAATGGAAGAAGCAAGTAAATGGTTAGAACGATATCAAGGTACATAG
S6_c.252A>T         CTTGAAGAATTTGATAAACAGGAAATAAAAGAAATGGAAGAAGCAAGTAAATGGTTAGAACGATATCAAGGTACATAG
S6_c.491A>G         CTTGAAGAATTTGATAAACAGGAAATAAAAGAAATGGAAGAAGCAAGTAAATGGTTAGAACGATATCAAGGTACATAG
```
