# Supplementary material for: The 3-phosphoinositide–dependent protein kinase 1 is an essential upstream activator of protein kinase A in malaria parasites
Source: PLoS Biol. 2021 Dec 8;19(12):e3001483. doi: 10.1371/journal.pbio.3001483 (PMC8687544; doi:10.1371/journal.pbio.3001483)
Supplement: Attachment — Submitted filename: Author response letter_final.pdf [file pbio.3001483.s019.pdf]

Response to Reviewers (Hitz et al.)

We thank the editors and reviewers for their fair and critical assessment and important input on our manuscript and for the invitation to submit a revised manuscript.

Editorial requests

Dear Dr. Voss,

Thank you very much for submitting your manuscript "The 3-phosphoinositide-dependent protein kinase 1 is an essential upstream activator of protein kinase A in malaria parasites" for consideration as a Research Article at PLOS Biology. Your manuscript has been evaluated by the PLOS Biology editors, an Academic Editor with relevant expertise, and by several independent reviewers.

In light of the reviews (below), we are pleased to offer you the opportunity to address the comments from the reviewers in a revised version that we anticipate should not take you very long. We will then assess your revised manuscript and your response to the reviewers' comments and we may consult the reviewers again.
>>>We thank the editorial team for this favourable decision and are delighted to submit a revised version of our manuscript.

In particular, we are overruling reviewer #1's first set of concerns on the rigidity issue, but we think it is important that you address reviewer #1's concern on whether the two proteins truly interact in vivo. Reviewer #1 suggests several experiments, one of which you could do to demonstrate direct interaction, or by discussing the caveat for your conclusion as pointed out by the reviewer. Please also address the comments the reviewers make on the writing and clarifications on the text.
>>>We addressed all editorial requests and reviewer comments (please see below).

Reviewer #1:

This is a really interesting manuscript investigating the role of the catalytic subunit of the cAMP-dependent protein kinase (PKAc) in Plasmodium falciparum asexual blood stage development and sexual development as well as its regulation. The study uses a number of approaches including conditional overexpression and conditional knockdown to confirm the role of PKAc in erythrocyte invasion, but also to show no apparent essential role in gametocyte commitment or development. The central hypothesis of the study is that PKAc is activated by an orthologue of the 3-phosphoinositide-dependent protein kinase 1 (PDK1). Overall the manuscript represents a huge amount of work involving many excellent experiments carried out very carefully and that are very well presented. The manuscript is also very nicely written and includes helpful summary statements in the individual results sections keeping the reader on board throughout. My additional thoughts and concerns are detailed below.
>>>We thank reviewer 1 for her/his appreciation of our work.

Figure 1. As the authors point out, there is previous literature implicating cAMP/PKA in gametocyte deformability and so I think that these experiments were a good idea. However, the differences in gametocyte retention using microsphiltration +/- Shield 1 shown in the plot, seem very small. Visually they appear non-significant, although it is stated that the differences are significant using an unpaired two-tailed Student's t test ($p = 0.04$). The SD values for stage V gametocytes seem quite large. It would be nice to see how big the differences in retention are using a 'positive control' (with WT gametocytes) that has been shown previously to change gametocyte retention significantly. This would help to determine whether the observed level of change is comparable with what has been found previously. I

note that only one biological replicate (with 6 technical replicates) of the control experiment with WT gametoctyes +/- Shield 1 has been presented which doesn't increase confidence in the this part of the work. As it stands it is hard to judge the biological relevance of these observations with such a small difference in retention upon depletion of such a key signalling molecule. I think without additional experiments to increase confidence in this part of the work, the authors should consider removing the data from the manuscript.

>>>We agree with reviewer 1 that knockdown of PfPKAc expression resulted only in a slight increase in gametocyte deformability. However, we observed this effect for both immature and mature gametocytes (Fig. 1E). Furthermore, our findings are consistent with the results from several previously published studies that presented indirect or direct evidence for a role for cAMP/PfPKA signaling in the regulation of gametocyte deformability. We therefore prefer keeping these data in the manuscript. We interpreted these data very cautiously and stated in the manuscript that while our findings suggest that PfPKAc contributes to the regulation of gametocyte deformability, it does not appear to be a main driver of this process. We now also wrote "…slight but significant decrease" instead of "significant decrease" in the Results section.

The control data obtained with WT gametocytes are shown in Fig. S4F. They confirm the typical increase in deformability of mature compared to immature gametocytes (showing that the assay worked properly) and that Shield-1 treatment has no effect on deformability of WT gametocytes for both stages (showing that the increase in deformability observed for the PfPKAc-depleted gametocytes was not due to Shield-1 treatment but rather to the reduction in PfPKAc expression levels).

Overexpression of an ectopic PKAc driven by a constitutive promoter was lethal and gave rise to parasites which stalled at the ~2 nuclei early schizont stage. Importantly the timing of this overexpression phenotype is very different to that reported previously using conditional knockout that showed a role for PKAc in erythrocyte invasion. However, conditional knockdown of PKAc confirmed its role in invasion. Interestingly, in the PKAc overexpression experiment, parasites reappeared in cultures again after 2 weeks; they still over-expressed PKAc and there was no mutation in either the ectopic or endogenous PKAc. These parasites showed normal nuclei numbers and blood stage development.

It is really intriguing that in the parasites selected, that could tolerate overexpression of PKAc, WGS showed mutations only in the putative PDK1 gene. (At the top of page 9, why are the M1 and M2 clones referred to as 'unselected clones'? I think that the term is confusing and should be deleted and the clones just referred to as M1 and M2 that have been described earlier in the text).

>>>We agree with reviewer 1 and replaced "unselected NF54/PKAc cOE clones M1 and M2" with "parental NF54/PKAc cOE clones M1 and M2".

The homology model of the PfPDK1 orthologue predicts nicely where the mutant residues are situated and how they are thought to affect catalytic activity, but not interaction with other proteins.

No PDK1 null could be obtained, but the conditional knockdown was non-essential suggesting that residual protein was sufficient for parasite development.

Does the PDK1 knockout/knockdown work add anything to the paper, especially because the results of the two approaches taken are conflicting? The inability to generate a null mutant suggests that PDK1 is essential in blood stages consistent with cited published work whereas the knockdown shows no phenotype, leading the authors to conclude that residual protein allows the parasite to develop normally. However, the level of knockdown (Figure 4b) looks very good to me. Could the findings of these experiments be just mentioned in the Discussion rather than presenting the results? Do the authors have any thoughts on why the multiplication rate is higher in the knockdown parasites?

>>>While we were unable to detect any obvious effects on asexual and sexual stage viability upon the conditional knockdown of PfPDK1 expression, we believe these data are still important enough to be included in the manuscript. First, this cell line allowed us to monitor PfPDK1 expression and localisation in asexual parasites. Second, this cell line may be

suitable for future experiments to study PfPDK1 function, for instance by performing transcriptomics or phosphoproteomics experiments. Third, our results indicate that future experiments aiming to study PfPDK1 loss-of-function phenotypes will likely require the generation of a DiCre-inducible KO line. Unfortunately, we have no explanation for the slightly increased multiplication rate of PfPDK1 knockdown parasites.

The main issue I have with the manuscript is as follows. On the one hand if PDK1 is indeed a physiological regulator of PKAc in P. falciparum, this has been a fantastic approach leading to its elucidation that will surely be a good approach for other researchers to follow in future to identify regulatory proteins. On the other hand unfortunately, it seems plausible that selection of these parasites with PDK1 mutations has taken place simply because they can survive the lethal effects of this artificial PKAc overexpression that does not occur at this life cycle stage in WT parasites and that the PDK1 orthologue is not actually a regulator of PKAc.
>>>We respectfully disagree with this comment. Even though the OE of PfPKAc occurs prior to the expression of endogenous PfPKAc, our results demonstrate that parasites can only survive PfPKAc OE by mutating PfPDK1, and we observed this six times independently. While these observations do not unequivocally prove that PfPDK1 directly phosphorylates/activates PfPKAc, they do demonstrate that PfPDK1 regulates PfPKAc activity, either directly or indirectly. We confirmed these findings by (1) introducing the PfPDK1 M51R mutation discovered in PfPKAc S1 OE survivor into the parental M1 clone; (2) reverting the M51R mutation in the S1 OE survivor back to the PfPDK1 WT sequence; and (3) by showing that OE of a non-phosphorylatable PfPKAc T189V mutant kinase was well tolerated in parasites expressing WT PfPDK1 and did not result in the selection of PfPDK1 mutations. This set of data clearly demonstrates that PfPDK1 is a physiological regulator of PfPKAc activity, irrespective of the fact that PfPKAc was artificially overexpressed. Furthermore, our data are entirely consistent with the evolutionary conserved role for PDK1 in binding to and activating PKAc through activation loop phosphorylation. Hence, in our opinion, there is no reason to believe, and no evidence to suggest, that PfPDK1 only regulates the activity of overexpressed PfPKAc but not endogenous PfPKAc.

Therefore I don't agree with the statement (on page 12 under the subheading) : 'To confirm the specific function of PfPDK1 in regulating PfPKAc activity…etc'. This is because in this section the authors further demonstrate the importance of the PDK1 M51R mutation in mediating parasite survival of the PfPKAc-GFP overexpression line by generating additional transgenic lines. In the summary of this section of the results on page 13, there is a more cautious statement that I do agree with: '…suggest that PfPDK1 is the kinase that phosphorylates and activates PfPKAc'.
>>>We changed the sentence on page 15 (line 401) "To confirm the specific function of PfPDK1 in regulating PfPKAc activity…" to "To test if PfPDK1 is indeed involved in regulating PfPKAc activity…".

Even though the presence (and amplification) of PDK1 mutations in the survivor parasites is compelling, what is lacking in the manuscript is an experiment that shows activation of PKAc by PDK1 or their mutual interaction in wild type parasites. This would be needed to provide direct evidence that PDK1 is a physiological regulator of PKAc and to dismiss the nagging suspicion/possibility that this may be an artificial survival mechanism in parasites selected due to overexpression of PKAc at the wrong* life cycle stage (*the essential role of PKA is in invasion, the lethal phenotype here is in early schizonts). Whilst I agree that the results strongly imply that PKAc and PDR1 should be able to interact in WT parasites, the question is: do they actually ever interact with each other in WT parasites?
Regarding demonstration of the interaction, I am really not a believer in the value of showing phosphorylation of recombinant proteins by a recombinant kinase as I think the results of this type of experiment can be misleading because in vitro some kinases will phosphorylate any protein with a consensus substrate site. This would give no information on whether the two proteins are normally expressed at the same time and in the same place. It would be great if

a pull down or e.g. a BioID approach was able to demonstrate a direct interaction between PKAc and PDK1 in both WT and survivor mutant parasites to help provide this missing link. Another experiment that would increase confidence in the hypothesis that PDK1 is a physiological PKAc activator is to try to complement the PKAcDD (endogenous locus) knockdown line with an ectopic copy of PKAc (under the control of the PKAc promoter) that carries the T189V substitution (in parallel with a control experiment adding an ectopic WT copy). If the hypothesis is correct it should not be possible to achieve complementation with the T189V copy as PKD1 would not be able to activate this mutant PKAc and invasion would not be possible.

>>> We demonstrate that (1) six out of six independently selected PfPKAc OE-tolerant mutants carry missense mutations in the PfPDK1 catalytic domain; (2) targeted mutagenesis of PfPDK1 renders parasites tolerant to PfPKAc OE; (3) reversal of the M51R PfPDK1 mutation renders parasites sensitive to PfPKAc OE; (4) parasites expressing WT PfPDK1 are tolerant to OE of the non-phosphorylatable PfPKAc-T189V activation loop mutant; (5) PfPKAc and PfPDK1 are both expressed in the cytosol in schizont stage parasites; and (6) mutating PfPDK1 was only possible in PfPKAc OE parasites, but not in WT parasites that express normal PfPKAc levels. Collectively, this conclusive set of data clearly demonstrates that PfPKAc activity is PfPDK1-dependent, irrespective of the fact that PfPKAc was artificially overexpressed. Given that PDK1 is known to interact with and phosphorylate PKAc in other eukaryotes, it is therefore only logical to assume that this interaction is also conserved in P. falciparum. We do agree with reviewer 1 that further experiments would be required to formally confirm this interaction in vivo, and we now explicitly mention this in the Discussion section on page 23 (line 605). However, identifying kinase-substrate interactions is generally very difficult as they are often transient and not robust enough to be reliably detected. We anticipate we would need at least 6-12 months to design, optimise and conduct informative co-IP or BioID experiments. To perform co-IPs followed by Western blot we would need to engineer a new cell line expressing both endogenous proteins as tagged versions. Co-IP with tagged PfPDK1 followed by mass spectrometry would require pilot experiments to optimise conditions and subsequent biological triplicate experiments to obtain reliable data. BioID experiments would require the generation of new transgenic cell lines and the clever design of adequate control conditions to deal with the large number of false-positive hits that are usually obtained using in vivo biotinylation approaches. Furthermore, any failure to detect an interaction between PfPDK1 and PfKAc in such experiments would not provide solid proof for a true lack of an interaction between these two factors in vivo. We therefore believe it is outside the scope of our already very comprehensive and convincing study to perform such time- and resource-intensive and challenging experiments, simply to confirm that PDK1 interacts with and phosphorylates PKAc also in malaria parasites as expected. However, we think it is an excellent idea to perform PfPDK1 co-IP and/or BioID experiments in the future as an exciting entry point into a PfPDK1-centered follow-up study aiming to understand the role of PfPDK1 in parasite biology in much greater detail.

The second experiment suggested by reviewer 1 is also interesting, yet very challenging as it would require the insertion of a single expression cassette into an ectopic locus (which is difficult to achieve) and would rely on the proper activity of the pfpkac promoter also at the ectopic location (which is not guaranteed). While the proposed experiments would allow to further address the importance of the T189 residue in PfPKAc activity, we believe they would still not demonstrate that T189 is phosphorylated by PfPDK1.

Even though the survivor population are fully viable, if mutant PDK1 is less able to phosphorylate/activate PKAc, you might expect a reduced erythrocyte invasion efficiency (since PKAc is essential for invasion) in the survivor parasites. Has the efficiency of invasion been measured in survivor parasites compared to WT to check this possibility? Any difference to WT would be interesting as it would be a link between mutant PDK1 and invasion.

>>>We measured almost identical parasite multiplication rates for M1/M2 parasites (wt PfPDK1) and S1 parasites (mutant PfPDK1), showing that RBC invasion is not impaired in parasites expressing mutant PfPDK1 (Figs. 2C, D, F, G and S6D).

I note that PDK1 is a relatively small kinase (525 amino acids). It would be nice to know in future how the mutations affect catalytic activity (as hypothesised) compared to WT. Perhaps kinase assays could easily be done on immunoprecipitated existing tagged WT and mutant PDK1 isoforms?
>>>We agree that such experiments would be informative to learn more about PfPDK1 function in the future. This would require the generation of new transgenic cell lines expressing tagged wt and mutant PDK1 and the establishment of a suitable substrate for in vitro kinase assays.

A very nice experiment is carried out showing that overexpression of a non-phosphorylatable Thr/Val substitution in the PKAc activation loop (which in other species is the target of PDK1) was not lethal. This provides further confirmation that PKAc and the PDK1 orthologue interact in the overexpression lines. I have to say that although this is not the wild type situation, manipulation of this specific threonine residue to reverse the lethal phenotype of PKAc overexpression does add an extra layer of confidence that the two proteins are likely to be physiological partners.
>>>We thank reviewer 1 for this comment.

Finally the activity (EC50 ~1 µM) of three inhibitors of human PDK1 on blood stage P. falciparum multiplication is demonstrated. However it would be useful if the authors could determine/clarify the stage at which these inhibitors are killing blood stage parasites to confirm whether or not this is consistent with PDK1 being their primary target. Either way I agree that PDK1 is worthy of additional future attention as a potential antimalarial drug target.
>>>We performed additional drug assays. First, we tested these inhibitors against PfPDK1-depleted parasites using our NF54/PDK1 cKD line. We expected to observe a reduced IC_{50} value for PfPDK1-depleted parasites if these inhibitors were indeed specific for PfPDK1. However, we found no difference in IC_{50} valus for NF54/PDK1 cKD parasites cultured in the presence (control) or absence of Shield-1 (PfPDK1-depleted). These new results are presented in Fig. S14 B and suggest that all three inhibitors target additional essential kinases. We also assessed parasite morphology in drug-treated parasites. These new results are shown in Fig. S14 C and D and suggest that all three inhibitors kill all intra-erythrocytic stages.

In the Discussion, the first statement should be modified to reflect the strong possibility that phosphorylation of proteins additional to AMA1 will underlie the essential role of PfPKA.
>>>We changed this sentence accordingly: "PfPKA is essential for the proliferation of asexual blood stage parasites, and the phosphorylation of the invasion ligand AMA1 is one of its crucial functions"

The statement: '..we discovered the upstream kinase that is required for PfPKAc activation' seems a little strong since no direct evidence of an interaction between PKAc and PDK1 has been demonstrated in WT parasites. The statement should be softened to reflect the fact there is a degree of uncertainty as I have argued above.
>>>We changed this sentence accordingly: "… we discovered another kinase that is likely required for PfPKAc activation."

Similarly, the later statement (top of page 18) beginning: 'Together these striking results demonstrate…'. I think the demonstrate should be changed to strongly suggest, but I agree that the results 'provide compelling evidence that PfPDK1 is the kinase that targets this residue'.
>>>We now wrote "imply" instead of "demonstrate".

Similarly, the later statement (top page 19): 'we identified PfPDK1 as the upstream kinase activating PfPKAc…'.

>>>We now wrote "…we provide compelling evidence that PfPDK1 is required to activate PfPKAc, …".

Similarly at the end of the Introduction (page 5) 'and that activation of PfPKAc is strictly dependent on PfPDK1-mediated phosphorylation'.
>>>We now wrote "'…and that activation of PfPKAc is likely dependent on PfPDK1-mediated phosphorylation".

Also the at the end of the Abstract: 'and identify PfPDK1 as a crucial upstream regulator in this pathway'.
>>>We now wrote "…and imply that PfPDK1 acts as a crucial upstream regulator in this pathway".

All of these statements should be softened.
>>>We softened all these statements (see above).

One thing that is surprisingly missing from the manuscript is any mention/discussion of how cAMP levels might impact the PfPDK1 activation process. I presume that the authors are thinking that activation of the overexpressed PKAc can happen independently of cAMP. I think that if there is insufficient PKAr expressed at this stage to form complexes with the excess PKAc, this could indeed be the case. However, in WT parasites, cAMP is obviously needed to activate PKAc (by binding to PKAr and causing dissociation of the complex allowing PKAc activation). I think this issue should be briefly dealt with in the Discussion. It will be interesting in future to explore the dynamics of the process by which PKAc is activated in the presence of cAMP and PDK1 in WT parasites, if it indeed turns out to be an essential upstream PKAc activator as the experiments in this study have strongly suggested.
>>>Yes, this is what we believe is happening in the PfPKAc OE line. We now mention this specifically in the Discussion section on page 21 (line 582): "We believe this detrimental effect is *due to the incapacity of endogenous PfPKAr to complex and inactivate the excess of PfPKAc enzymes*, resulting in illegitimate activity of free PfPKAc and hence untimely phosphorylation of substrates prior to the intrinsic PfPKA activity window in late schizonts."). We already stated in the Discussion section that cAMP is needed in WT parasites to release and activate PKAc (page 22, line 603): "Together, these striking results imply that in addition to the cAMP-mediated release of PfPKAc from the regulatory subunit PfPKAr, phosphorylation of the T189 residue is essential for PfPKAc activity, and provide compelling evidence that PfPDK1 is the kinase that targets this residue."

<u>Reviewer #2:</u>

The manuscript by Hitz et al uses elegant malaria genetic manipulations to understand the role of PfPKA catalytic domain in the sexual stages of the malaria parasites. While the authors find that PfPKAc does not play a major role in sexual commitment/gametocyte maturation, it does contribute to gametocyte rigidity (though possibly not the major driver). Using an overexpression system of PfPKAc, the exciting result is the "survivor" parasites have only compensatory mutations in PfPDK1 using whole genome sequencing, suggesting that a regulation network between these two kinases. The authors follow up on this premise with further predicted targeted selected mutations in genetically modified parasites to show that T189 in the PfPKAc activation loop is crucial. As with PfPKA, the authors show that PfPDK1 is also not involved in gametocyte/sexual commitment.

While the authors did not uncover a new function for both these kinases in gametocyte function, this paper represents a substantial amount of work that is extremely thorough and well validated. It is biologically relevant as all the work was performed in parasites vs recombinant kinases. In fact, it is quite hard to pick any further experiments or repeats that I would have like to see or required further interpretation. The authors should also be

commended on not over stating any result that they have, especially around the gametocyte rigidity. In addition, while somewhat aside to their main results, they describe an important caveat using GlnN in studying sexual commitment and male gametocyte exflagellation.
>>>We thank Dr. Wai-Hong Tham very much for her entirely positive assessment of our manuscript.

My only minor comment is for the authors to perhaps provide a rationale on why they made the M51R transgenic versus any of the other mutations?
>>>We chose M51R simply because mutating the corresponding codon using CRISPR/Cas9 was most straightforward and promising for this residue.
